# Sequence division and coal facies research methods of the deep No. 8 coal seam in the Daning-Jixian Block, eastern margin of Ordos basin

**DOI:** 10.1038/s41598-025-27709-z

**Published:** 2025-12-18

**Authors:** Jintao Kong, Xianyue Xiong, Rong Ding

**Affiliations:** 1 China United Coalbed Methane National Engineering Research Center Company Limited, Beijing, 100095 China; 2 PetroChina Coalbed Methane Company Limited, Beijing, 100028 China

**Keywords:** Eastern margin of the ordos basin, Sequence stratigraphy, Coal facies, Sparrow search algorithm (SSA), Support vector regression (SVR), K-means cluster, Energy science and technology, Engineering, Environmental sciences, Solid Earth sciences

## Abstract

With the large-scale commercial development of deep coalbed methane in the Ordos Basin, the refined research on deep coal seams (with a burial depth > 2000 m) has begun to attract much attention. In this study, the No. 8 coal seam of the Taiyuan Formation in the Daning-Jixian Block on the eastern margin of the Ordos Basin was taken as the research object. By combining sequence stratigraphy theory and coal facies research methods, and using the Support Vector Regression (SVR) model optimized by the Sparrow Search Algorithm (SSA) as well as the clustering algorithm, SSA-SVR models based on logging parameters for calculating the vitrinite—inertinite ratio (V/I ratio) and the Texture Preservation Index (TPI) value, along with a coal facies division scheme suitable for the Daning-Jixian Block, was established. The results demonstrate that the unbiased Mean Absolute Percentage Error (MAPE) of the V/I-SSA-SVR model is 18.73%, the unbiased Root Mean Square Error (RMSE) is 1.36, and the unbiased Coefficient of Determination (R²) is 88.69%. For the TPI - SSA - SVR model, the unbiased MAPE is 17.90%, the unbiased RMSE is 1.12, and the unbiased R² is 86.39%.Through clustering analysis of nearly 5000 sets of V/I ratios and TPI values calculated by SSA-SVR models in the study area, The TPI values and V/I ratios were each divided into four categories. By combining these two parameters, a classification scheme for coal facies research in the Daning-Jixian Block was finally developed. Based on the modeling results, this study proposed a sequence stratigraphy division method for the deep No. 8 coal seam in the Daning-Jixian Block. By using the V/I-SSA-SVR model, the vertical V/I ratios of the No. 8 coal seam in individual wells was calculated. Combined with lithological changes, the water depth transition interfaces were identified. Based on this method, the Daning-Jixian Block can be divided into 5 fifth-order sequences. Furthermore, by combining the TPI-SSA-SVR model and the established coal facies classification scheme, this study finally reconstructed the coal-forming environments of each fifth-order sequence during the formation of the No. 8 coal seam in the Daning-Jixian Block. For the first time, this study conducted refined research on China’s first commercial breakthrough block for deep coalbed methane, filling the gap in the research on deep coal sequences and coal facies. It has established coal facies parameter boundary values and a division scheme exclusive to the deep No. 8 coal seam in the Daning-Jixian Block, innovatively proposed the “V/I-SSA-SVR sequence division method”, and provided guidance and reference for subsequent exploration and development of deep coalbed methane.

**Supplementary Information:**

The online version contains supplementary material available at 10.1038/s41598-025-27709-z.

## Introduction

Since the 1980 s, with the vigorous development of sequence stratigraphy theory, an increasing number of coalfield geologists have begun to apply sequence stratigraphy to the study of coal-bearing strata^1–8^. Most studies usually regard widely distributed and stably developed coal seams as a marker horizon in the sedimentary process, and generally focus on the division of third-order sequences and their coal-controlling effect in the entire basin^9–11^. However, with the large-scale commercial development of deep coalbed methane in the eastern margin of the Ordos Basin in recent years^12,13^, refined research on deep coal seams (with a burial depth of > 2000 m) has begun to attract significant attention^14,15^.

In the field of oil and gas exploration and development, sequence stratigraphy plays an extremely important role. Based on sequence stratigraphy, researchers can establish a cyclic and genetically related chronostratigraphic framework^16^. By studying the sedimentary environment of each part in the chronostratigraphic framework and its associated evolutionary laws, it is possible to predict favorable hydrocarbon-bearing intervals, identify favorable reservoirs, and clarify the spatial-temporal distribution and configuration of various hydrocarbon accumulation elements^17–19^. Therefore, applying sequence stratigraphy to conduct high-frequency and short-cycle sequence division of coal seams with a thickness of 1 to 10 m, establishing a chronostratigraphic framework, and analyzing the sedimentary environment of each coal sublayer will lay a solid foundation for the study of deep coalbed methane accumulation mechanisms, and possess profound significance and important scientific implications for the exploration and development of deep coalbed methane.

As a relatively new type of reservoir, although there is as yet no systematic method for attempting sequence division within coal seams, the academic community has already developed a unique methodological framework for researching coal seam sedimentary environments. In 1951, the Soviet scholar Zhemchuzhnikov coined the term “coal facies” in his work *Methods for the Study of Coal Series*,* Coal Seams*,* and Coal*, using it to refer to the sedimentary environment of coal seams^20^. Over more than 70 years of development, research on coal facies has gradually evolved and matured. In simple terms, coal facies refers to the original genetic type of coal, which is determined by the environment in which peat formed coal facies research can provide information such as the coal-forming plants, peat accumulation environment, and coalification process of coal seams^21–26^. It enables a systematic understanding of coal-forming environments and lays the groundwork for further research^27,28^. At present, there are diverse research methods for coal facies, including coal petrology^29,30^, coal facies parameters^22,31–36^, and inorganic geochemistry^37–42^, among others.

Among the various methods, the coal facies parameter research method is particularly favored by researchers. This method determines coal facies by calculating quantitative values and then comparing them with established boundary values. However, due to geological heterogeneity and the great diversity of coal-forming environments, it seems unreasonable to apply a unified set of global coal facies parameters boundary values. Simply dividing coal facies by comparing with existing coal facies parameters can lead to unreliable and unscientific results.

This study takes the deep No. 8 coal seam in the Daning-Jixian Block on the eastern margin of the Ordos Basin as the research object. Its objectives are to explore high-frequency, short-cycle sequence division methods within the coal seam, establish a sequence stratigraphic framework for the deep No. 8 coal seam in the Daning-Jixian Block, and on this basis, determine coal facies parameter boundary values that can guide the development of deep coalbed methane in the Daning-Jixian Block. Additionally, it aims to conduct coal facies research based on the sequence stratigraphic framework, thereby providing scientific decision support for the subsequent exploitation of deep coalbed methane in this region. Different from previous studies, the innovations of this study are reflected in three aspects: First, it is the first time to conduct refined research on the Daning-Jixian Block, China’s first commercial breakthrough block for deep coalbed methane, filling the gap in the research on deep coal sequences and coal facies. Second, based on nearly 5000 sets of vitrinite-inertinite ratios (V/I ratios) and Texture Preservation Index (TPI) values calculated using the SSA-SVR model in the block, combined with K-means clustering, coal facies parameter boundary values and a division scheme exclusive to the deep No. 8 coal seam in the Daning-Jixian Block have been established. This avoids the limitations of general standards, which have coarse classification dimensions and cannot meet the precision requirements of coal facies for the refined development of deep coalbed methane. Third, the “V/I-SSA-SVR sequence division method” is innovatively proposed. By combining exclusive coal facies standards to reconstruct the coal-forming environment of each microsequence (fifth-order sequence), this study provides direct theoretical basis and technical pathways for the exploration and development of similar deep coalbed methane reservoirs.

## Geological setting

**Fig. 1:**
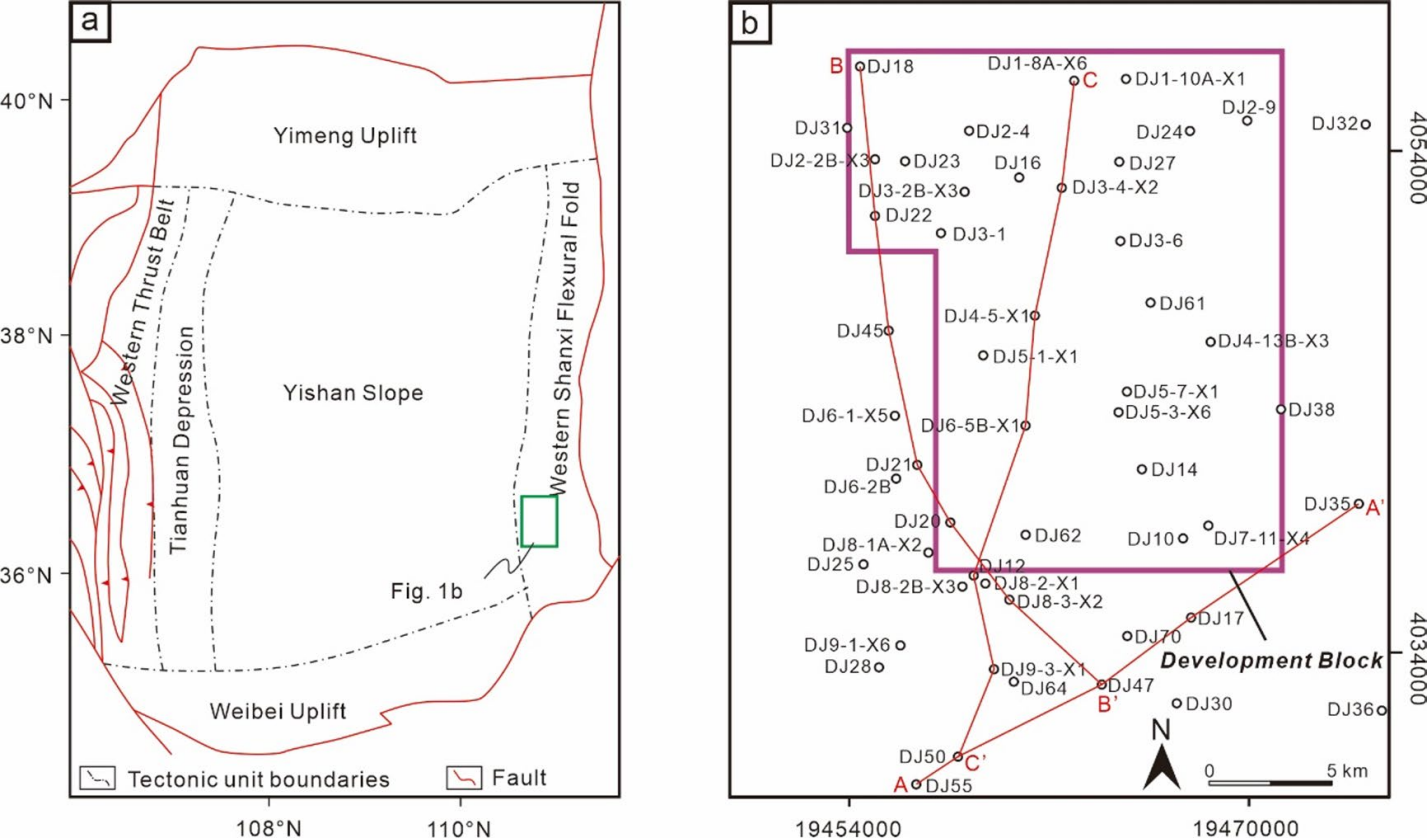
Regional geological maps. (**a**) Geological schematic map of the Ordos Basin. (**b**) Well location map of the Daning-Jixian Block.

The Ordos Basin is a large intracontinental foreland basin developed by the superimposition of Mesozoic and Cenozoic basins on the Paleoproterozoic North China Craton epicontinental platform^43,44^ (Fig. 1a). It is the not only the sedimentary basin with the earliest formation history and the longest evolutionary duration in China, but also the second largest onshore sedimentary basin in the country. The basin is bounded by the Yellow River Fault to the north, Uda-Pingliang to the west, Qishan and Jinhua Mountains to the south, and the Lüliang Mountains to the east. It comprises six major structural units, including the Western Shanxi Fold Belt, Yishan Slope, Tianhuan Depression, Western Thrust Belt, Weibei Uplift, and Yimeng Uplift^45,46^. The basement of the Ordos Basin consists of Neoarchean Tonalite–Trondhjemite–Granodiorite (TTG) rocks, gneiss, as well as Paleoproterozoic metamorphic rocks and volcanic rocks^47,48^. Overlying the basement are the sedimentary covers of the Mesoproterozoic Changcheng System, Jixian System, Paleozoic, Mesozoic, and Cenozoic. The sedimentary history of the basin exhibits a binary structure of marine and terrestrial facies^49–51^. During the Cambrian–Ordovician period, the basin began to form, primarily developing marine carbonate clastic formations^52,53^. From the Late Ordovician to the Early Carboniferous, affected by the Caledonian orogeny, the basin experienced uplift and erosion. Subsequently, during the Middle-Late Carboniferous, marine-continental transitional facies deposits were developed^54,55^. During the Permian–Jurassic periods, seawater completely retreated from the basin, and a set of continental clastic formations of fluvial-deltaic-lacustrine facies were developed within the basin^56–58^. Entering the Cenozoic, influenced by the collision between the Indian and Eurasian plates, as well as the subduction of the Pacific plate, multi-stage uneven uplift within the Ordos Basin, forming a reworked and remnant intra-cratonic basin^59,60^.

**Fig. 2:**
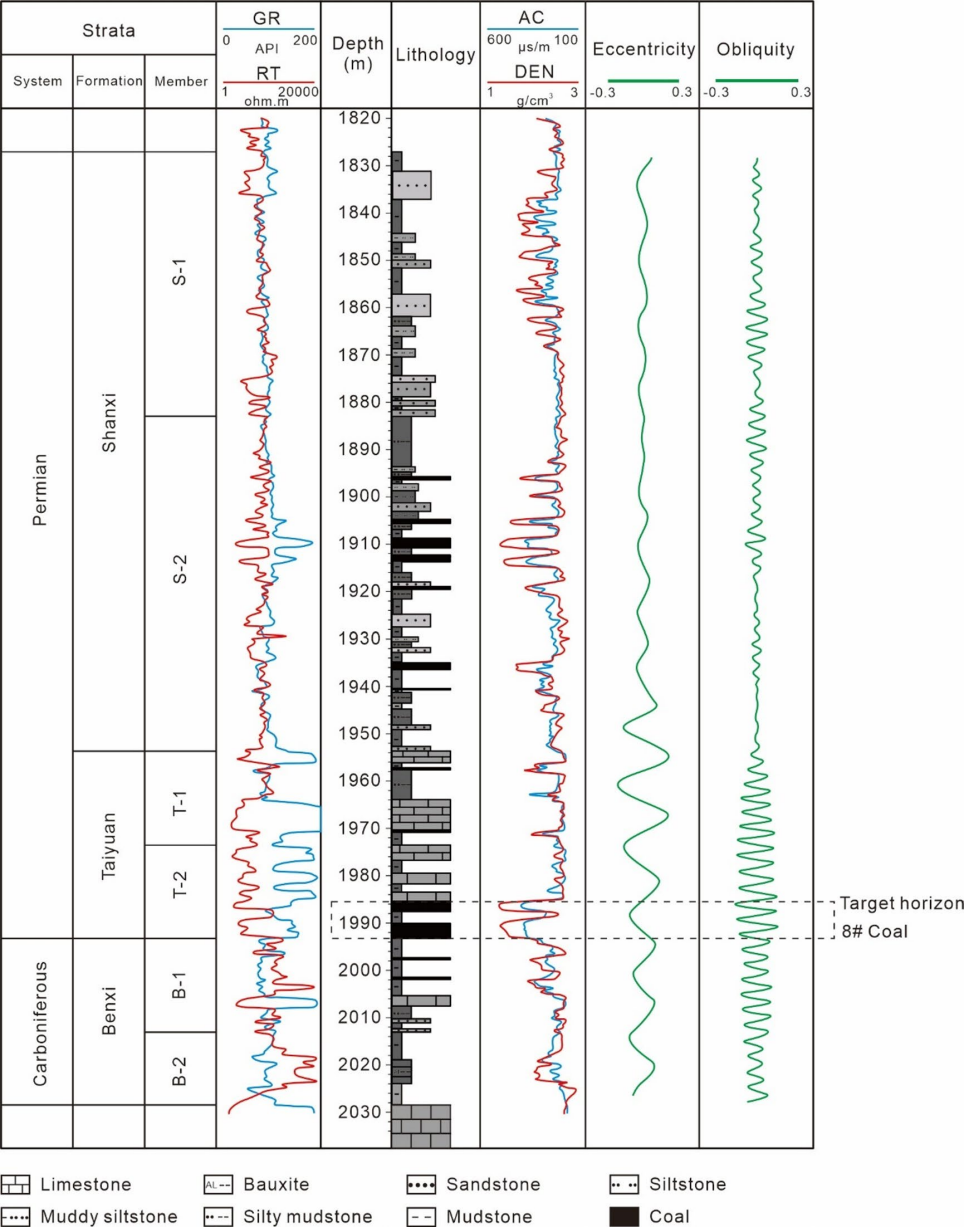
Comprehensive stratigraphic lithological columnar section of the typical well DJ70 in the Daning-Jixian Block.

The Daning-Jixian block is located on the eastern margin of the Ordos Basin (Fig. 1b). Regional drilling data indicate that the stratigraphic sequences, from oldest to youngest, consists of the Archean Shushui Group, Paleozoic Cambrian, Ordovician, Carboniferous, and Permian systems, Mesozoic Triassic and Cenozoic Quaternary (Fig. 2). Among these, the deep No. 8 coal seam is located at the base of the Permian Taiyuan Formation.

The Archean constitutes the basement rock of the study area, composed of augen granite gneiss and chlorite schist, with magnetite quartz veins. The Cambrian and Ordovician Majiagou Formation are dominated by marine carbonate rocks. The Carboniferous Benxi Formation is a set of coastal to shallow marine sedimentary formation, primarily consisting of limestone, sandstone, and mudstone. The Permian stratigraphy comprises the Taiyuan Formation, Shanxi Formation, and Shihezi Formation.

The Taiyuan Formation is continuously deposited over the Benxi Formation, representing a set of marine-continental transitional facies deposits. It has a thickness ranging from 40 to 60 m and is primarily composed of medium to fine-grained sandstone, mudstone, limestone, and coal seams-making it one of the major coal-bearing strata in this region. The formation contains 1–3 coal seams, numbered as No. 6, No. 7, and No. 8. Among these, the No. 8 coal seam has a thickness ranging from 4 to 11 m, with a vitrinite reflectance (Ro) ranging from 2.14 to 2.78% (average: 2.59%), classifying it as high-rank coal. It is distributed throughout the entire area and serves as a regional marker horizon.

Based on lithological, lothofacies, and sedimentary cycle characteristics, the Taiyuan Formation is divided into two members from bottom to top. The thickness of the Taiyuan 2 (Tai2) Member typically ranges from 10 to 30 m, and its lithology is dominated by bioclastic limestone, micritic limestone, and mudstone. From bottom to top, the limestones within this member are the Miaogou Limestone and Mao’ergou Limestone, which thin gradually toward the south. The No. 8 coal seam developed at the base is the main mineable coal seam, distributed throughout the entire area, serving as a regional marker horizon, and forms a conformable contact with the underlying strata. The thickness of the Taiyuan 1(Tai1) Member generally ranges from 5 to 30 m with lithology dominated by bioclastic limestone, micritic limestone, mudstone, and coal seams. From bottom to top, the limestones here are the Xiedao Limestone and Dongdayao Limestone. The No. 7 coal seam or associated carbonaceous mudstone develops above the Xiedao Limestone, while the No. 6 coal seam or carbonaceous mudstone develops at the top of the Dongdayao Limestone.

The Shanxi Formation consists of a set of marine-continental transitional facies. Its upper boundary is the bottom sandstone of the lower Shihezi Formation (commonly referred to as the Luotuobozi Sandstone), and its lower boundary is the regionally stably distributed No. 6 coal seam or carbonaceous mudstone. The upper part of the Shanxi Formation is characterized by well-developed sandstones, while the lower part features well-developed coal seams-with the development of No. 3, No. 4, and No. 5 mineable coal seams as its key trait, intercalated with deltaic subaqueous distributary channel sand bodies. The formation has a thickness ranging from 80 to 160 m. Combined with sedimentary cycle characteristics and electrical logging markers, it is divided into two members from bottom to top. The Shanxi 2 (Shan2) Member has a thickness of 65–90 m, consisting of a set of fluvial-deltaic coal-bearing deposits composed of medium- to fine-grained sandstones interbedded with mudstones and coal seams. The Shanxi 1 (Shan1) Member is 35–60 m thick, representing subaqueous distributary channel sand and mudstone deposits.

The Shihezi Formation is divided into the Lower Shihezi Formation and Upper Shihezi Formation. The Lower Shihezi Formation comprises interbedded layers of gray mudstone and light-colored fine sandstone with unequal thicknesses, intercalated with sandy mudstones, and fine sandstone develops at its base. The Upper Shihezi Formation is dominated by sandstone at the base, mudstone interbedded with fine sandstone in the lower part, and fine sandstone interbedded with mudstone in the upper section.

The Triassic strata include the Liujiagou Formation, Heshanggou Formation, and Zhifang Formation, which are widely exposed in the main river valleys of the Yellow River tributaries within the study area. These formations exhibit a complete stratigraphic sequence, simple structural and continuous deposition, with lithology dominated by fine sandstone and mudstone. The Quaternary strata directly overlie strata of various geological ages, with lithology mainly consisting of light yellow to yellow silty soils and red subclay.

## Methodology

### Scheme for sequence hierarchy division

Rational division of sequence hierachy is a fundamental issue in sequence stratigraphy. Based on years of research, geologists have proposed two schemes for sequence hierarchy division. The first scheme classifies sequence into hierarchies according to the characteristics and attributes of sequence boundaries, without being restricted by time scales^61–65^. This scheme has strong practicality and facilitates sequence hierarchy division in small isolated continental basins. However, it has two obvious shortcomings. First, it emphasizes that tectonic activity is the controlling factor in sequence formation, which leads to contradictory conclusions when classifying sequences formed by sea-level fluctuations. Second, it has high requirements for the preservation degree of strata at basin margins, resulting in significant limitations in application^66,67^.

The second scheme is time-based and takes global sea-level changes as the main driving force for the formation of sequence stratigraphic units at all hierarchies^68–74^. Emerging with the advent of sequence stratigraphy, this scheme has been widely applied and gained recognition and support from numerous scholars. Nevertheless, it also has certain drawbacks. Due to temporal and spatial differences in research objects and subjectivity in the understanding of sequence genetic mechanisms, different scholars hold varying views on the time scales of sequences at different hierarchies. Considering that the sequence hierarchy division scheme proposed by Wang and Shi^75^ was summarized based on practical cases from Himalayas, Tarim Basin, and North China Craton, and is consistent with China’s geological background, this study adopts the scheme proposed by Wang and Shi^75^.

This scheme classifies sequence hierarchies into five levels: megasequence, mesosequence, orthosequence, subsequence, and microsequence. Currently, sequence stratigraphic division of coal-bearing strata focuses primarily on the orthosequence level, which refers to a sedimentary sequence formed within a single third-order sea-level change cycle, with thickness ranging from 40 to 150 m and an average of approximately 120 m^75^. Sedimentary sequences at this level hold particularly significant importance in sequence stratigraphic studies and are regarded as the basic stratigraphic unit of sequence stratigraphy^68,71,76^. A subsequence is a secondary sedimentary sequence within an orthosequences. Mitchum et al.^71^ proposed that the magnitude of fourth-order sea-level changes is approximately 20 m; thus, the thickness of subsequences ranges from several meters to tens of meters, with a duration of about 0.1 to 0.4 Ma and an average duration of approximately 0.3 Ma. This is largely consistent with the Milankovitch eccentricity cycle (0.1–0.4 Ma)^77,78^.

A microsequence is the smallest identifiable sedimentary sequence in sequence stratigraphy. Wang and Shi^75^ argued that the sea-level change for a fifth-order sequence (i.e., a microsequence) is approximately 15 m. Numerous studies have shown that the thickness of microsequences varies from tens of centimeters to several meters, with an average duration of approximately 0.02–0.04 Ma, which aligns with the precession and obliquity cycles of the Milankovitch cycles^71,78,79^.

The floor of the No. 8 coal seam in the study area consists of tidal flat deposits within a lagoonal environment. These deposits were exposed and underwent pedogenesis during a regression event, belonging to root-soil deposits with developed plant roots. During the seawater regression, incomplete withdrawal of seawater left the basin in a shallow-water environment, allowing plants to spread and peat swamps to develop. Geochemical characteristics of the No. 8 coal seam indicate that the paleo-salinity of the coal seam is even higher than that of its roof limestone^80^. This fully demonstrates that during the development of peat swamps, seawater frequently intruded into the basin interior. Minor fluctuations in sea-level influenced the development of peat swamps, until episodic transgressive events in the later stage terminated peat swamp development, leading to coalification of peat swamps under deep-water conditions. Our goal is to divide microsequences (fifth-order sequences) within the No. 8 coal seam. Recently, the research team conducted astronomical cycle analysis on Well DJ70 in the block using paleoclimatic proxies. Based on long eccentricity and obliquity filtering curves, high-resolution fourth-order and fifth-order sequence stratigraphic frameworks were established for the block (in press). The results show that five half-cycles were identified in the obliquity filtering curve of the No. 8 coal seam interval, confirming that the No. 8 coal seam in the Daning-Jixian Block has a geological basis for fifth-order sequence division. However, this method relies on astronomical signals recorded in the entire stratigraphic sequence for fifth-order sequence division. Due to variations in the overall sedimentary environment of the strata, its precision cannot meet the requirements for fine division within the coal seam, with relatively large errors. Therefore, there is an urgent need to further explore a high-precision fifth-order sequence division scheme suitable for the interior of coal seams.

The sequence hierarchy division scheme proposed by Wang and Shi^75^, adopted in this study, inherits from the Exxon School, which holds that sequence formation is closely related to sea-level changes. For microsequences (fifth-order sequences), they reflect fifth-order cycles of relative sea-level fluctuations. To finely divide fifth-order sequences within the No. 8 coal seam, thereby identifying transition surfaces corresponding to the water depth shallowing-deepening cycle process, which serve as sequence boundaries between individual microsequences (fifth-order sequences).

### SSA-SVR coal facies parameters models based on logging responses

In coal facies research, coal facies parameters are primarily classified into two categories: (1) Quantitative parameters reflecting water depth, such as Gelification Index (GI)^22^, Groundwater Influence Index (GWI)^33^, Wetness Index (WI)^34^, and Vitrinite - Inertinite Ratio (V/I, Harvey and Dillon, 1985; Yuan and Li, 2015)^31,81^. (2) Quantitative parameters reflecting vegetation types, such as the Tissue Preservation Index (TPI)^22^, Vegetation Index (VI)^33^, and Tissue Index (TI)^34^.

**Fig. 3:**
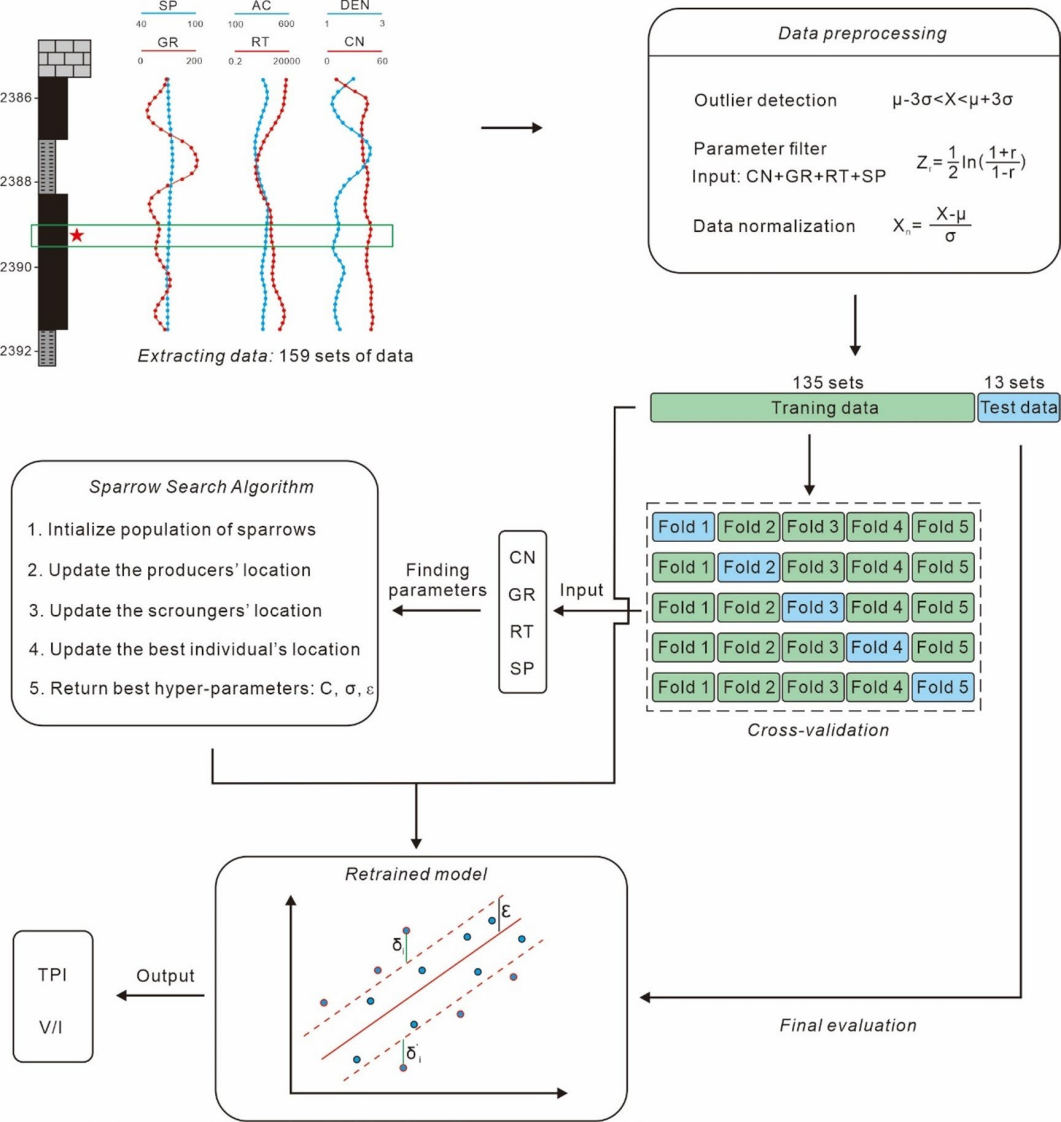
Schematic diagram of the research workflow for coal facies parameter models based on logging responses.

Coal facies parameters are calculated based on the maceral content within coal. However, the current analytical and test data of coal rocks in the Daning-Jixian Block are limited, which severely restricts the study of coal facies. To address this issue, we extracted the corresponding conventional logging parameters—including Resistivity (RT), Gamma Ray (GR), Acoustic (AC), Density (DEN), Neutron (CN), and Spontaneous Potential (SP)—from coal rock samples obtained from drilling that had undergone maceral analysis. We then selected an appropriate machine learning algorithm to establish a coal facies parameter models based on logging responses, which are used to calculate coal facies parameters for coal rocks without maceral analysis in the Daning-Jixian Block (Fig. 3).

In this study, based on the measured data of 67 coal rock samples, a total of 159 maceral content datasets containing logging data were constructed through core orientation, data integration, and matching. Using this dataset (Supplemental Data 1), two SSA-SVM models for calculating TPI values and V/I ratios were established, and models were used to conduct a systematic analysis of 47 wells in the study block.

#### Petrological mechanism of logging responses

Conventional logging parameters contain abundant geological and stratigraphic information. Since different geological reservoirs exhibit unique logging response characteristics, logging technology can effectively address geological and reservoir evaluation issues^82,83^. The maceral components in coal seams exhibit significant differences in structural characteristics, and their different proportions result in differences in petrophysical information and logging response characteristics. To analyze the logging response characteristics corresponding to different TPI values and V/I ratios, the Pearson correlation coefficient was first used to analyze the correlation between each conventional logging parameter and the TPI values/V/I ratios of coal seams; meanwhile, the correlation coefficients between the logging parameters themselves were calculated (Table 1):

$$\:r=\frac{\sum\:_{i=1}^{n}({x}_{i}-\bar{x})({y}_{i}-\bar{y})}{\sqrt{\sum\:_{i=1}^{n}{\left({x}_{i}-\bar{x}\right)}^{2}\sum\:_{i=1}^{n}{\left({y}_{i}-\bar{y}\right)}^{2}}}$$

**Table 1:** Correlation coefficients between TPI, V/I values and logging parameters.

	TPI	V/I	AC	CN	DEN	GR	RT	SP
TPI	1.000							
V/I	0.340	1.000						
AC	−0.069	0.118	1.000					
CN	0.167	0.140	0.460	1.000				
DEN	0.040	– 0.133	– 0.384	0.029	1.000			
GR	– 0.146	– 0.168	– 0.332	– 0.084	0.723	1.000		
RT	0.144	0.111	– 0.041	– 0.128	– 0.076	0.000	1.000	
SP	0.106	– 0.212	0.058	0.127	0.405	0.114	– 0.125	1.000

Parameter optimization is essential for logging parameter-based modeling. Correlations between logging parameters significantly impact model performance, and excessive redundant information is likely to introduce noise into the model. A z-test based on the Pearson correlation coefficient was used to determine the independence of logging parameters^84^. The null hypothesis $\:{{H}_{0}:\:r}_{0}\left(x,y\right)=0$ was established, and under this null hypothesis, the test statistic was constructed as follows:

$$\:{z}_{r}=\frac{1}{2}\text{ln}\:(\frac{1+r}{1-r})\sim N\left(\frac{1}{2}\text{ln}\left(\frac{1+{r}_{0}}{1-{r}_{0}}\right),\frac{1}{\sqrt{n-3}}\right)$$

Under the null hypothesis, when the significance level α = 0.05, the critical value is 0.132. The $\:{z}_{r}$ statistics between each pair of logging parameters were calculated separately (Table 2). The results show that AC is not independent of CN, DEN, or GR; DEN is not independent of GR or SP. Therefore, in the final model construction, only CN, GR, RT, and SP were selected as input parameters in this study. Additionally, the TPI values and V/I ratios exhibit correlations of varying degrees with these four logging parameters. TPI is the ratio of structured plant tissues to unstructured fine plant fragments in coal. The degree of destruction of plant tissues affects the physical properties of coal rocks, which in turn results in significant differences in logging responses. The specific relationships are as follows:

During the coalification process of structured plant tissues, the organic matter is relatively orderly arranged, with more primary cell cavities retained—this leads to high gas content and weak electrical conductivity of the coal seam. In contrast, unstructured fine fragments have a loose structure and disordered pores, resulting in low gas content and enhanced electrical conductivity. Therefore, the higher the TPI, the higher the coal RT.

Coal seams containing abundant structured plant tissues have more developed pores and higher coalbed methane content. CN reflects the hydrogen content in the formation; thus, the higher the TPI, the higher the response value of CN.

The SP in coal seams mainly originates from the electromotive force of redox reactions: it exhibits a positive anomaly in oxidizing environments and a negative anomaly in reducing environments. Diessel et al.^82^ pointed out that TPI can reflect the degree of oxidation—coal formed under weakly oxidizing conditions has a higher TPI, while coal formed under dry oxygen or extremely humid conditions has a lower TPI. Therefore, the lower the TPI, the more obvious the SP anomaly.

GR reflects the content of radioactive elements in the formation, and its response to coal depends primarily on the content of clay minerals. Although clay minerals are not involved in TPI calculation, plant debris may be mixed with clay minerals during transportation, which indirectly affects GR. A higher content of clay minerals indicates that the plant debris was transported over longer distances, leading to a relatively lower TPI.

V/I is the ratio of vitrinite content to inertinite content in coal seams. In general, inertinite is formed by fusainization under conditions of shallow water cover, dryness, and oxidation in swamps; whereas vitrinite is formed by gelification in environments with closed air circulation and relatively deep water cover. The significant differences in their physical properties result in a clear correlation between V/I values and logging response characteristics:

Vitrinite has more developed pores and fractures (such as primary cell cavities and cleats) and contains more coalbed methane, leading to a high response value in CN logging. Thus, V/I values exhibit a positive correlation with CN.

Vitrinite forms in reducing environments with water cover, containing little mixed clay minerals—hence resulting in a lower GR value. In contrast, inertinite forms in oxidizing environments, which are prone to associated clay mineral deposition, leading to a higher GR value. Additionally, the incorporation of clay minerals in coal seams reduces RT. Therefore, a higher V/I value corresponds to lower clay mineral content, a smaller GR value, and a larger RT value.

Vitrinite forms in reducing environments, where the SP shows a negative anomaly; inertinite forms in oxidizing environments, where SP shows a positive anomaly. Consequently, V/I values exhibit a negative correlation with SP.

**Table 2:** Statistic $\:{\varvec{z}}_{\varvec{r}}$ calculated based on pearson correlation coefficients among various conventional logging parameters.

	AC	CN	DEN	GR	RT	SP
AC						
CN	0.49					
DEN	0.39	0.028				
GR	0.34	0.083	0.907			
RT	0.04	0.128	0.07	0.0001		
SP	0.05	0.127	0.42	0.113	0.125	

#### Algorithm introduction

Compared to other machine learning models, the Support Vector Regression (SVR) model offers advantages, such as strong generalization ability, no local minima, and effective handling of nonlinear features^85,86^. Most importantly, this model can effectively solve learning problems with small sample sizes^87^. This method creates a “margin band” on both sides of a linear function, with a spacing of 2ε (where ε is referred to as the tolerance margin). No loss is calculated for all samples falling within this margin band. Finally, the optimized model is obtained by minimizing the total loss and maximizing the margin. In practical tasks, it is often difficult to directly determine an appropriate ε that ensures most data points fall within the margin band. Similar to SVM, we introduce slack variables $\:{\delta\:}_{i}$ and $\:{{\delta\:}_{i}}^{*}$, where $\:{\delta\:}_{i}>0$ and $\:{{\delta\:}_{i}}^{*}=0$ above the margin band, and $\:{{\delta\:}_{i}}^{*}>0$ and $\:{\delta\:}_{i}=0$ below the margin band. When dealing with complex nonlinear regression, we map the vector $\:\overrightarrow{x}$ to a high-dimensional space $\:\phi\:\left(\overrightarrow{x}\right)$ and introduce a kernel function $\:K\left({x}_{1},{x}_{2}\right)$. At this point, the regression equation is:

$$\begin{aligned}f\left(\overrightarrow{x}\right)&={\overrightarrow{\omega\:}}^{T}\phi\:\left(\overrightarrow{x}\right)+b\\ & \quad =\sum\limits_{i=1}^{N}{\alpha\:}_{i}{y}_{i}K\left(\overrightarrow{x},\overrightarrow{{x}_{i}}\right)+b\end{aligned}$$

Our objective is:

$$\:{min}_{\overrightarrow{\varvec{\omega\:}},\:{\delta\:}_{i},\:{{\delta\:}_{i}}^{*}}\frac{1}{2}{||\overrightarrow{\omega\:}||}^{2}+C\sum\limits_{i=1}^{N}({\delta\:}_{i}+{{\delta\:}_{i}}^{*})$$

$$\:\varvec{s}.\varvec{t}.\:\left\{\begin{array}{c}{y}_{i}-f\left(\overrightarrow{{x}_{i}}\right)\le\:\epsilon\:+{\delta\:}_{i}\\\:{f\left(\overrightarrow{{x}_{i}}\right)-y}_{i}\le\:\epsilon\:+{{\delta\:}_{i}}^{*}\\\:{\delta\:}_{i},{{\delta\:}_{i}}^{*}\ge\:0\end{array}\right.$$

Where $\:C$ is the regularization parameter, which controls the complexity of the model. The aforementioned optimization problem is converted into a dual problem, which is then optimized using the Sequential Minimal Optimization (SMO) algorithm. In this study, a Gaussian kernel function was used to construct the SVR model. The hyperparameters in this case include the regularization parameter $\:C$, the scaling parameter $\:\sigma\:$ of the Gaussian kernel function, and the tolerance margin $\:\epsilon\:$.

To ensure the rationality of hyperparameter selection in the model, the Sparrow Search Algorithm (SSA) was adopted in this study for determining the model’s hyperparameters. The SSA, developed by Xue and Shen^88^, is a novel intelligent optimization algorithm inspired by the foraging and anti-predation behaviors of sparrows. Owing to its strong optimization capabilities, rapid convergence speed, and global search ability, this algorithm has been widely applied in the determination of hyperparameters in machine learning and deep learning.

To describe the SSA algorithm in mathematical terms, a sparrow population can be represented as:

$$\:X=\left[\begin{array}{c}\begin{array}{ccc}{x}_{1}^{1}&\:{x}_{1}^{2}&\:\begin{array}{cc}\cdots\:&\:{x}_{1}^{d}\end{array}\end{array}\\\:\begin{array}{ccc}{x}_{2}^{1}&\:{x}_{2}^{2}&\:\begin{array}{cc}\cdots\:&\:{x}_{2}^{d}\end{array}\end{array}\\\:\cdots\:\\\:\begin{array}{ccc}{x}_{n}^{1}&\:{x}_{n}^{2}&\:\begin{array}{cc}\cdots\:&\:{x}_{n}^{d}\end{array}\end{array}\end{array}\right]$$

where *d* is the dimension of the optimization problem, and *n* is the number of sparrows. The fitness of all sparrows can be expressed as:

$$\:{Fitness}_{x}=\left[\begin{array}{c}f\left({x}_{1}^{1}\:\:{x}_{1}^{2}\:\:\:\cdots\:\:\:\:{x}_{1}^{d}\right)\\\:f\left({x}_{2}^{1}\:\:{x}_{2}^{2}\:\:\:\cdots\:\:\:\:{x}_{2}^{d}\right)\\\:\cdots\:\\\:f\left({x}_{n}^{1}\:\:{x}_{n}^{2}\:\:\:\cdots\:\:\:\:{x}_{n}^{d}\right)\end{array}\right]$$

The update of the producer’s position is described as follows:

$$\:{X}_{i,\:j}^{t+1}=\left\{\begin{array}{c}{X}_{i,\:j}^{t}\bullet\:exp\left(\frac{-i}{\alpha\:\bullet\:{iter}_{max}}\right),\:\:if\:{R}_{2}<ST\\\:{X}_{i,\:j}^{t}+Q\bullet\:L\:\:\:\:\:\:\:\:\:,\:\:if\:{R}_{2}\ge\:ST\end{array}\right.$$

Here, *t* represent the current iteration number, and $\:{x}_{i,j}$ denote the position information of the *i*-th sparrow in the *j*-th dimension. The parameters $\:\alpha\:\in\:(0,\:1]$, $\:{R}_{2}\in\:[0,\:1]$ and $\:ST\in\:[0.5,\:1]$ correspond to the warning value and safety value, respectively. *Q* is a random number following a normal distribution. Additionally, *L* is a 1×d matrix, where all elements are 1. When $\:{R}_{2}$< ST, this indicates that there are no predators around, allowing the producer to perform extensive search operations. When $\:{R}_{2}$≥ST, it means that some sparrows in the population have detected predators and have issued an alarm to the other sparrows, prompting all sparrows to swiftly fly to other safe locations.

The position update formula for the scrounger is described as follows:

$$\:{X}_{i,\:j}^{t+1}=\left\{\begin{array}{c}Q\cdot\:exp\left(\frac{{X}_{worst}^{t}-{X}_{i,\:j}^{t}}{{i}^{2}}\right)\:\:\:\:\:\:\:\:\:\:,\:\:if\:i>\frac{n}{2}\\\:{X}_{P}^{t}+\left|{X}_{i,j}-{X}_{P}^{t}\right|\cdot\:{A}^{T}{\left(A{A}^{T}\right)}^{-1}\cdot\:L,\:\:if\:i\le\:\frac{n}{2}\end{array}\right.$$

$\:{X}_{p}$ represents the current optimal position occupied by the producer, and $\:{X}_{worst}$ is the current global worst position. A is a 1×d matrix, where each element is randomly assigned a value of 1 or −1. When i > n/2, it indicates that the *i*-th sparrow, which has a lower fitness value, has not obtained food. In this case, the sparrow needs to fly to another location to forage in order to gain more energy. Conversely, when i ≤ n/2, the scrounger s attempt to occupy the producer’s position to seize food.

The mathematical expression of the sparrows’ anti-predation behavior upon sensing danger can be expressed as:

$$\:{X}_{i,\:j}^{t+1}=\left\{\begin{array}{c}{X}_{best}^{t}+\beta\:\cdot\:\left|{X}_{i,\:j}^{t}-{X}_{best}^{t}\right|\:\:,\:\:if\:{f}_{i}>{f}_{g}\\\:{X}_{i,\:j}^{t}+K\cdot\:\left(\frac{\left|{X}_{i,\:j}^{t}-{X}_{worst}^{t}\right|}{\left({f}_{i}-{f}_{w}\right)+\epsilon\:}\right),\:\:if\:{f}_{i}={f}_{g}\end{array}\right.$$

$\:{X}_{best}$ represents the optimal position among all current parameters. $\:\beta\:\sim\:N\left(\text{0,1}\right)$ is a random variable controlling the step size. $\:K\in\:[-1,\:1]$ is a uniformly distributed random number. $\:{f}_{i}$ denotes the fitness value of the current individual sparrow. $\:{f}_{g}$ and $\:{f}_{w}$ are the current global best and worst fitness values, respectively. $\epsilon$ is an extremely small constant to avoid a zero denominator. When $\:{f}_{i}>{f}_{g}$, it indicates that the sparrow is situated at the edge of the population, extremely vulnerable to predator attacks, and needs to move toward the optimal position. When $\:{f}_{i}={f}_{g}$, it signifies that sparrows located in the center of the population have perceived danger and need to move a certain distance randomly to minimize the risk of being preyed upon. K represents both the direction of the sparrow’s movement and the step size control parameter.

Finally, a SVR model optimized by the SSA (SSA-SVR) based on logging responses was established to calculate the V/I ratios and TPI values.

To evaluate the performance of the constructed models, this study employed the Mean Absolute Percentage Error (MAPE), Root Mean Square Error (RMSE), and coefficient of determination (R²) as the models’ evaluation metrics.

$$\:MAPE=\frac{1}{n}\sum\limits_{i=1}^{n}\frac{\left|{y}_{i}-\widehat{{y}_{i}}\right|}{{y}_{i}}\times\:100\%$$

$$\:RMSE=\sqrt{\frac{1}{n}\sum\limits_{i=1}^{n}{(\widehat{{y}_{i}}-{y}_{i})}^{2}}$$

$$\:{R}^{2}=1-\frac{{\sum\:_{i=1}^{n}({y}_{i}-\widehat{{y}_{i}})}^{2}}{\sum\:_{i=1}^{n}{({y}_{i}-\bar{y})}^{2}}$$

### K-means clustering algorithm

After calculating the coal facies parameters, it is necessary to compare them with critical values to determine the specific coal-forming environment. Existing studies suggest the following criteria: a V/I ratio greater than 4 represents strong water cover; a V/I ratio between 1 and 4 indicates extremely humid-water covered conditions; a V/I ratio between 0.25 and 1 denotes humid to weakly water covered conditions; and a V/I ratio less than 0.25 indicates dry to extremely dry conditions with fire events^81,89^. For TPI values, a TPI greater than 1 suggests that the coal-forming swamp was dominated by ligneous plants and a TPI less than 1 implies that the coal-forming swamp was dominated by herbaceous plants^32^. However, as previously described, it is unscientific to apply a unified global standard to coal facies research. Drawing on the idea of coal facies parameter classification from previous studies, this research adopts an unsupervised learning method-K-means Clustering Algorithm-to determine the critical threshold values of V/I ratios and TPI values that are suitable for coalbed methane development in the Daning-Jixian Block.

The K-means Clustering Algorithm is an unsupervised learning method based on iterative solution, and it is widely used due to its simplicity, intuitiveness, and high efficiency^90^. The goal of this algorithm is to partition *n* data points into *K* clusters, such that each data point belongs to the cluster whose center is closest to it. By repeatedly adjusting the positions of cluster centers, the algorithm continuously optimizes intra-cluster compactness, thereby obtaining clusters that are as compact and well- separated from each other. The implementation steps of the K-means Clustering Algorithm are as follows:

Input: Dataset $\:D={x}_{1},{x}_{2},\dots\:\dots\:,{x}_{n}$, number of clusters *k*.

Output: Clustering results.

1. Initialization: Randomly initialize *k* samples as cluster centers $\:\left\{{\mu\:}_{1},{\mu\:}_{2},\dots\:\dots\:,{\mu\:}_{k}\right\}$;
2. Assignment: Calculate the distance $\:dist({x}_{i},\:{\mu\:}_{j})$ between each sample $\:{x}_{i}$ in the dataset and each cluster center $\:{\mu\:}_{j}$, and assign $\:{x}_{i}$ to the cluster where the closest cluster center is located.
  $$\:dist\left({x}_{i},\:{\mu\:}_{j}\right)=\sqrt{\sum\limits_{i=1}^{n}{\left({x}_{i}-\:{\mu\:}_{j}\right)}^{2}}$$
3. Update: for each cluster, update its cluster center as follows
  $$\:{\mu\:}_{j}=\frac{1}{\left|{c}_{j}\right|}\sum\limits_{x\in\:{c}_{j}}x$$
4. Iteration: Repeat Steps 2 and 3 until there is no significant change in the cluster or the preset number of iterations is reached.

The specific method for determining the critical values of the V/I ratios and TPI values is as follows:

Step 1: We employ the V/I-SSA-SVR and TPI-SSA-SVR to calculate the V/I ratios and TPI values of the No. 8 coal seam in 47 wells within the block. The logging data resolution of all wells if 0.125 m. Based on the average coal seam thickness of approximately 10 m, a single well can yield approximately 80 sets of V/I ratios and TPI values.

Step 2: The 3σ method was used to remove outliers from all obtained V/I ratios and TPI values, i.e., removing outliers that fall outside the interval $\:\left(\mu\:-3\sigma\:,\:\mu\:+3\sigma\:\right)$ (where $\:\mu\:$ is the mean and $\:\sigma\:$ is the standard deviation). Only the V/I ratios and TPI values within this interval were subjected to cluster analysis using the K-means Clustering Algorithm.

Step 3: Analyze the critical values between each cluster, and then determine the final critical values for V/I ratios and TPI values.

To determine the optimal K value (number of clusters) for TPI values and V/I ratios, this study adopted the elbow method. The Sum of Squared Errors (SSE) under different K values (number of clusters) was calculated, and a curve was plotted with K values as the horizontal axis and SSE as the vertical axis. In the curve, as K increases, SSE decreases gradually; when K increases to a certain extent, the rate of SSE decline slows down significantly, and the curve shows an inflection point similar to an “elbow.” The K value corresponding to this inflection point is the optimal number of clusters. This method is used to ensure that the clustering results not only well reflect the intrinsic distribution characteristics of the data but also avoid problems caused by excessive or insufficient clustering.

## Results

### Coal facies parameter calculation model

Before modeling, we performed preprocessing on 159 sets of data. First, the 3σ detection method was used to remove outliers: an input or output parameter value was identified as an outlier if it fell outside the µ ± 3σ interval. Finally, a total of 148 sets of data were retained. Due to the small sample size of this study, 5-fold cross-validation was adopted to obtain a stable and reliable model (Fig. 3). The specific steps are as follows:

Step 1: Divide the data into a training dataset (90% of the total data, 135 sets) and a test dataset (10% of the total data, 13 sets). Split the 135 training data samples into 5 folds, with each fold containing 27 samples.

Step 2: Set the number of iterations of the SSA algorithm to 11–30 and the maximum population size to 21–30 to optimize the model’s regularization parameter $\:C$, scaling parameter $\:\sigma\:$ in the Gaussian kernel function, and tolerance error $\:\epsilon\:$. For different combinations of iteration numbers and population sizes, perform 5 rounds of training and validation:

 1st round: Use folds 2–5 (108 samples in total) as training samples and fold 1 (27 samples) as validation data, and record the model evaluation metrics;

2nd round: Use folds 1, 3–5 (108 samples in total) as training samples and fold 2 (27 samples) as validation data, and record the model evaluation metrics;

3rd to 5th rounds: Repeat the process, using a different fold as validation data each time, resulting in 5 sets of validation metrics.

Step 3: Statistically summarize the 5 rounds of validation metrics, and calculate the mean (reflecting the model’s “average performance level”) and standard deviation (reflecting the model’s “stability”).

In model evaluation, the coefficient of determination R^2^ is the primary metric. First, models with large standard deviations are excluded; among the stable models, the one with the optimal performance is selected.

Step 4: After determining the hyperparameters of the optimal model, retrain the model using all training data (135 samples) to enable the model to learn the complete patterns. Then, evaluate the model’s generalization ability using the previously divided test dataset.

To eliminate the influence of dimensions, improve model convergence speed, and ensure the fairness of each input parameter, the data were subjected to standardization. The training set, validation set, and test set data are denoted as $\:{X}_{train},{Y}_{train},{X}_{valid},{Y}_{valid},{X}_{test},{Y}_{test},$ respectively. The means and standard deviations of the input and output parameters of the training set were calculated, denoted as $\:{X}_{mean},{Y}_{mean},{X}_{std},{Y}_{std}$, respectively. The standardized training set, validation set, and test set data are as follows:

$$\:{X}_{train}^{{\prime\:}}=({X}_{train}-{X}_{mean})/{X}_{std}$$

$$\:{Y}_{train}^{{\prime\:}}=({Y}_{train}-{Y}_{mean})/{Y}_{std}$$

$$\:{X}_{valid}^{{\prime\:}}=({X}_{valid}-{X}_{mean})/{X}_{std}$$

$$\:{Y}_{valid}^{{\prime\:}}=({Y}_{valid}-{Y}_{mean})/{Y}_{std}$$

$$\:{X}_{test}^{{\prime\:}}=({X}_{test}-{X}_{mean})/{X}_{std}$$

$$\:{Y}_{test}^{{\prime\:}}=({Y}_{test}-{Y}_{mean})/{Y}_{std}$$

Finally, 200 V/I-SSA-SVR models and 200 TPI-SSA-SVR models were evaluated respectively (evaluation metrics are provided in Supplemental Data 2). For the stable and optimal V/I-SSA-SVR model, the regularization parameter $\:C$ is 26.45, the scaling parameter $\:\sigma\:$ is 1.28, and the tolerance error $\:\epsilon\:$ is 0.4577. The unbiased MAPE, unbiased RMSE, and unbiased R^2^ calculated on the test dataset are 18.73%, 1.36, and 88.70%, respectively.

For the stable and optimal TPI-SSA-SVR model, the regularization parameter $\:C$ is 11.76, the scaling parameter $\:\sigma\:$ is 0.98, and the tolerance error $\:\epsilon\:$ is 0.7325. The unbiased MAPE, unbiased RMSE, and unbiased R^2^ calculated on the test dataset are 17.90%, 1.12, and 86.40%, respectively.

**Fig. 4:**
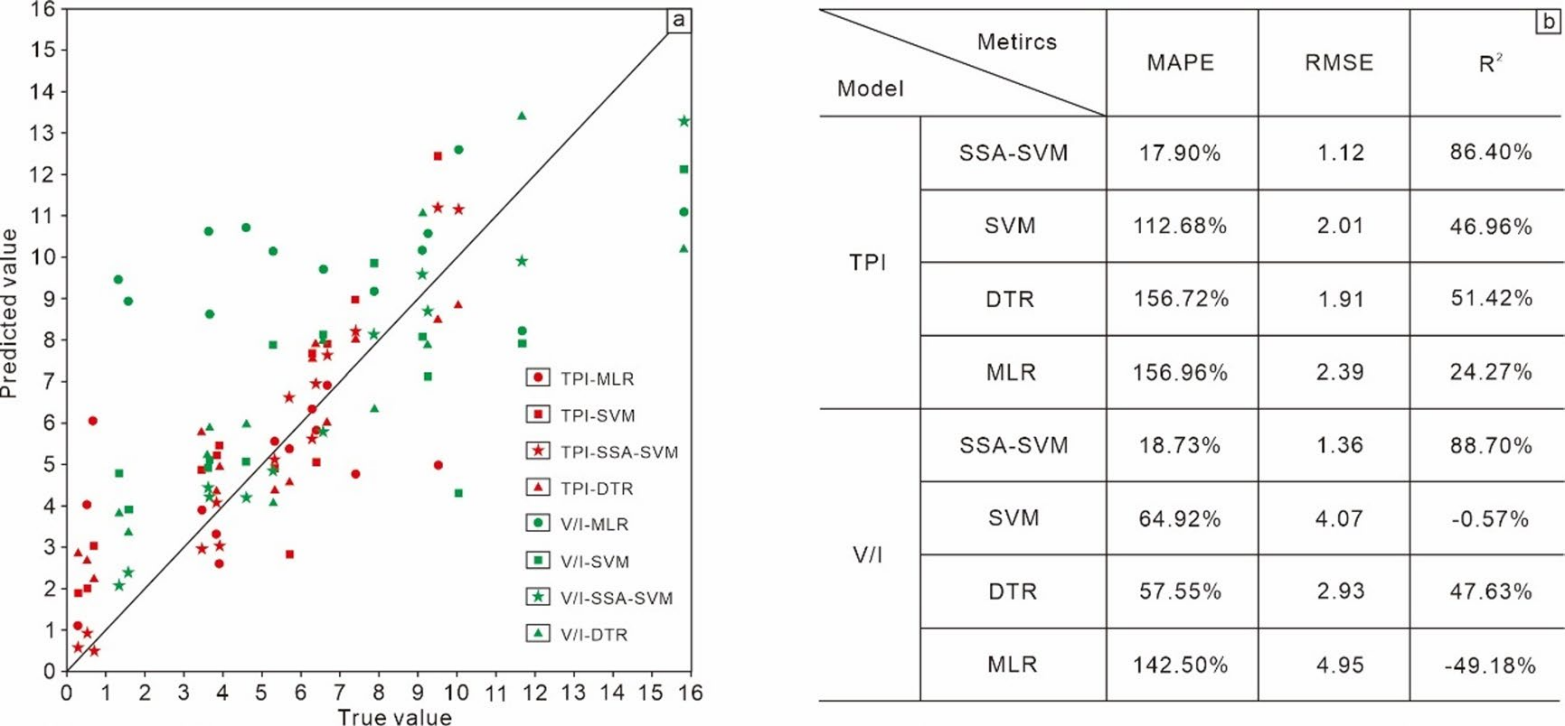
Cross-scatter plot of actual test values vs. model-predicted values for the test dataset (**a**) and prediction error indicators of different models (**b**).

By comparing algorithm models suitable for small-sample modeling, such as SSA-SVM, SVM, multiple linear regression, and decision tree regression, the results show that SSA-SVM performs the best in all metrics when predicting V/I ratios and TPI values, with prediction accuracy far superior to SVM, multiple linear regression, and decision tree regression (Fig. 4a and b).

### Coal facies parameter clustering results

**Fig. 5:**
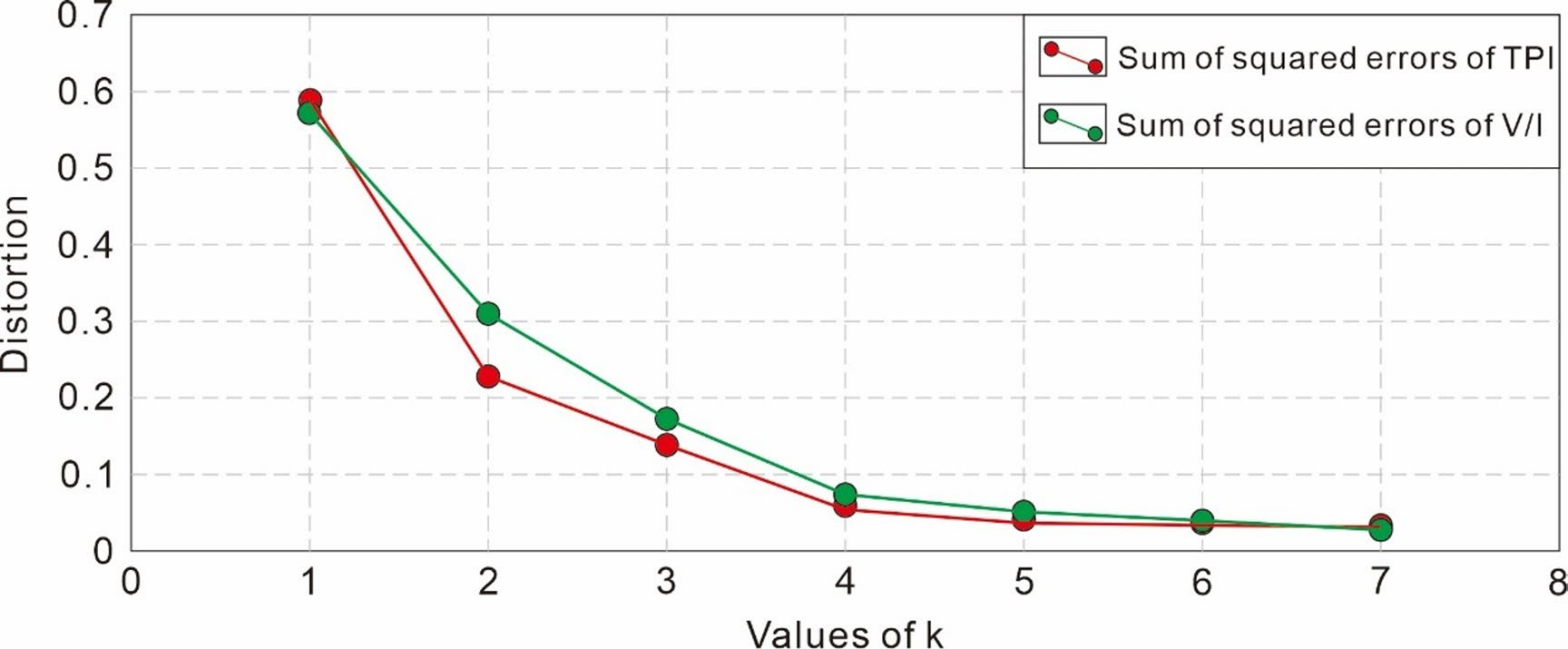
Line plot of Sum of Squared Errors (SSE) for TPI values and V/I values at different k values.

The V/I-SSA-SVR and TPI- SSA-SVR models were used to analyze the No. 8 coal seam encountered by 47 wells across the entire block, and a total of nearly 5000 sets of V/I ratios and TPI values were obtained. The 3σ principle was used to remove outliers, and more than 4400 sets of valid data were retained. Analysis via the elbow method showed that when the number of clusters for TPI values and V/I ratios was set to 4, the rate of SSE decline slowed down significantly (Fig. 5). Therefore, the optimal number of clusters for TPI values and V/I ratios was determined to be 4 in this study.

**Fig. 6:**
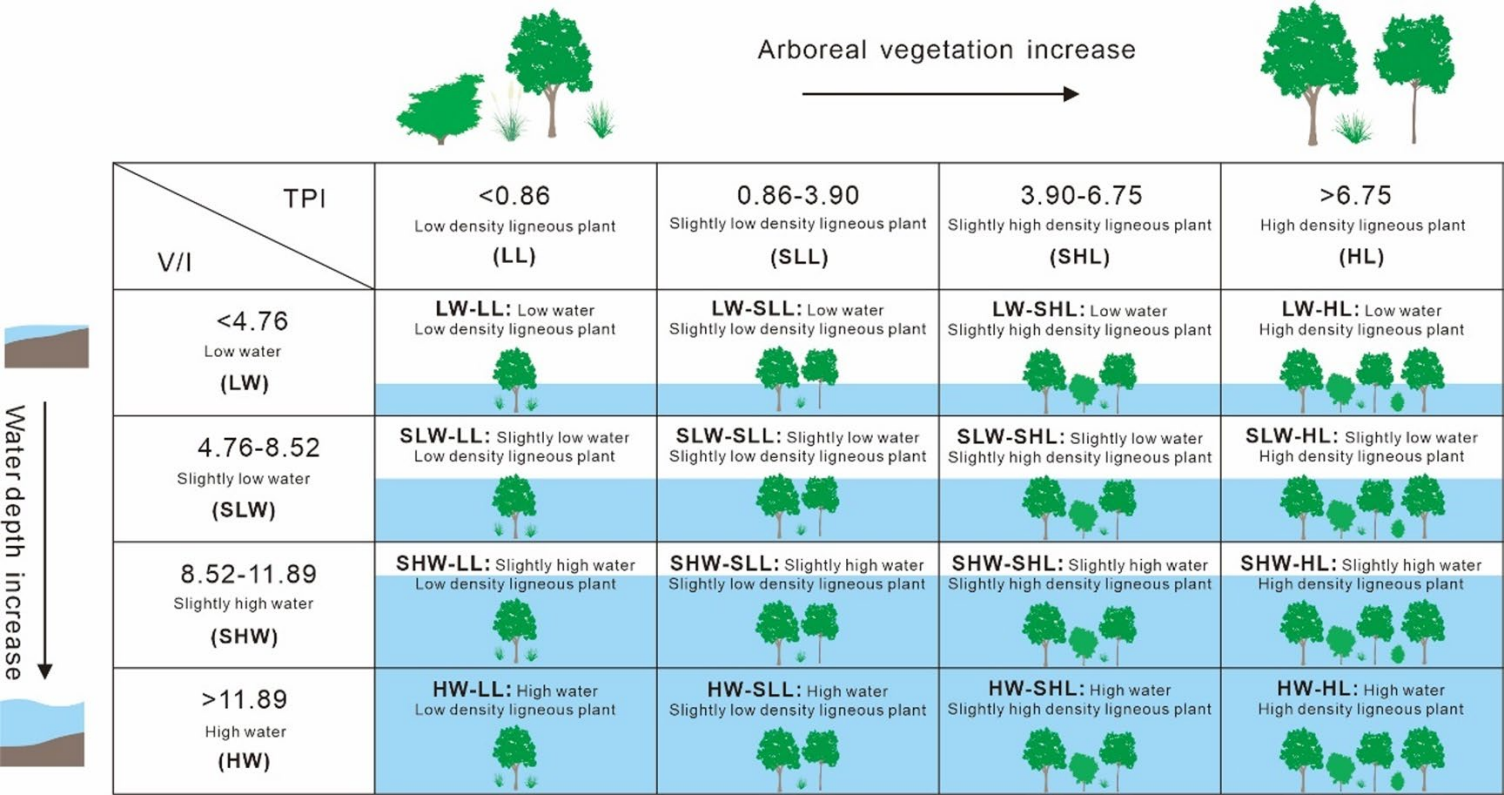
Classification scheme for coal facies in the Daning-Jixian Block.

According to previous studies, a higher V/I ratio indicates a deeper water depth in the coal-forming swamp^31^, and a higher TPI value indicates a higher ligneous plant density^22^. The indicative significance of each cluster of V/I ratios and TPI values was specifically elaborated (Fig. 6).

For TPI values: <0.86 indicates low-density ligneous plants, 0.86–3.90 indicates slightly low-density ligneous plants, 3.90–6.75 indicates slightly high-density ligneous plants, and > 6.75 indicates high-density ligneous plants.

For V/I ratios: <4.76 indicates low water depth, 4.76–8.52 indicates slightly low water depth, 8.52–11.89 indicates slightly high water depth, > 11.89 indicates high water depth.

It is important to emphasize that the critical values of coal facies parameters in this study are not absolute standards applicable to all regions and research scenarios. Their core scope of application is limited to the development analysis of the Daning-Jixian Block, and they are essentially relative reference values serving internal comparisons within this block.

Taking the V/I ratio as an example: if the V/I ratio of a certain area is less than 4.76, it cannot be directly determined that this area belongs to a shallow water environment universally applicable globally. Instead, it should be understood as follows: within the Daning-Jixian Block, the sedimentary water depth of this area is relatively shallower compared with other areas in this block, and this conclusion is only valid for horizontal comparisons within the Daning-Jixian Block. Similarly, for the TPI value, if the TPI value of a certain area is greater than 6.75, it does not mean that the development degree of ligneous plants in this area has reached an absolutely vigorous level. The correct interpretation is that the ligneous plants in this area are more vigorous compared with the development status of woody plants in other areas of the Daning-Jixian Block.

In short, the significance of these parameter critical values is limited to comparisons of differences within the Daning-Jixian Block. Beyond the scope of this block, the same values may not reflect the same geological characteristics. Therefore, in subsequent development and analysis, the application scenarios of these values must be strictly restricted to avoid misinterpreting relative values as absolute values.

By combining the critical values of V/I ratios and TPI values, 16 categories of coal facies classification criteria suitable for the Daning-Jixian Block were derived (Fig. 6).

## Discussion

### Single-well sequence stratigraphic analysis

Based on the V/I-SSA-SVR model developed in this study, the V/I ratio curves of the No. 8 coal seam in 47 wells were calculated to identify water depth transition interfaces. Additionally, since the V/I ratio is only applicable to coal rocks, water depth cannot be determined using the V/I cannot be determined using the V/I ratio for mudstone interlayers within the coal seams. However, the development of mudstone interlayers typically indicates that the rate of subaquatic accommodation space increase in much greater than the peat accumulation rate, signifying a sharp deepening of water depth. Thus, their top interfaces can be regarded as sequence boundaries.

Here, we take Well DJ62 as an example to elaborate on the specific division process. The No. 8 coal core of this well was scanned using a Philips Ingenuity full-diameter spiral CT scanner, and the scanning results can be used to verify the sequence division results.

Well DJ62 is located in the southern part of the study area. The entire No. 8 coal seam has a thickness of approximately 7.47 m and contains one mudstone interlayer with a thickness of about 0.7 m. During the division process, we defined the V/I ratio transition points within the coal seam and lithologic transition interfaces as sequence boundaries. Among these, the internal water depth transition interfaces within the coal seam are determined by V/I ratio inflection points, and we propose combining the visual observation method with the moving average method to identify V/I ratio inflection points.

The visual observation method involves approximating a trend line of V/I ratio changes based on the scatter plot of V/I ratios versus depth, and identifying the V/I ratio transition interfaces by observing the peaks and valleys of the curve. The moving average method judges the inflection points of V/I ratios by comparing the changing trends of moving averages before and after. The specific steps are as follows:

1. Select an appropriate window size $\:n$ ($\:n=5$ in this study) and calculate the moving average value $\:{MA}_{t}:$
  $$\:{MA}_{t}=\frac{{V/I}_{t-n+1}+{V/I}_{t-n+2}+\cdots\:+{V/I}_{t}}{n}$$.
2. Calculate the difference $\:\varDelta\:MA$
  $$\:\varDelta\:MA={MA}_{t}-{MA}_{t-1}$$

When the sign of $\:\varDelta\:MA$ changes, it indicates that $\:t$ is a potential inflection point. To avoid misjudgment caused by noise, a comprehensive judgement should be made in combination with the visual observation method.

**Fig. 7:**
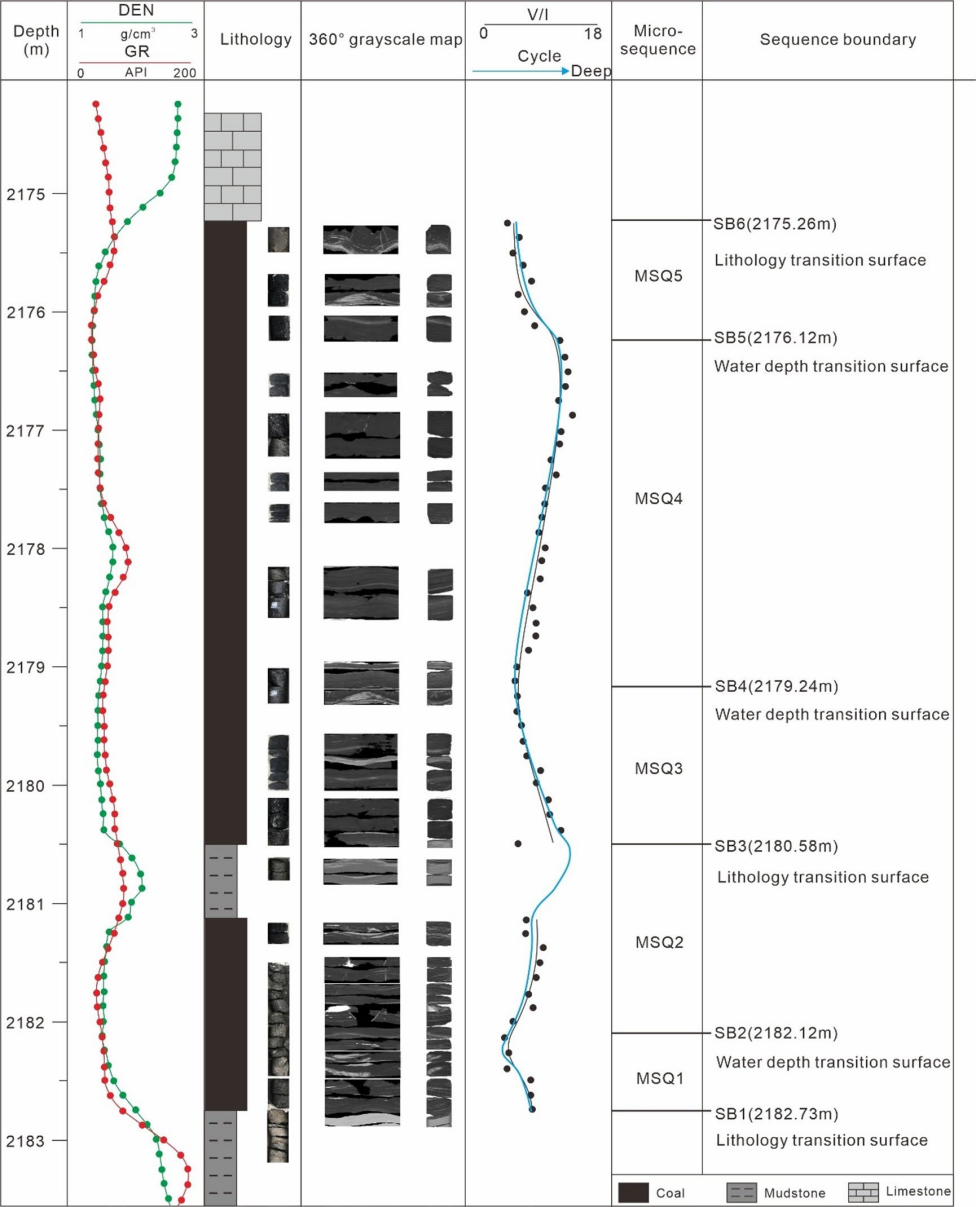
Microsequence (fifth-order sequence) division of the No. 8 coal seam in Well DJ62.

Based on the above principles, a total of 5 microsequences (fifth-order sequences) were divided in Well DJ62, which are MSQ1 to MSQ5 from bottom to top (Fig. 7).

The bottom boundary (SB1) of the MSQ1 is a lithologic transition surface from mudstone to coal seam, indicating a process of gradual retreat of water from the basin. Due to the incomplete retreat of seawater, the basin remained in a shallow water environment, and peat swamp development was initiated. The top boundary (SB2) of the MSQ1 is a transition interface indicating deepening water, as inferred from the V/I ratio. From the 3D grayscale model reconstructed from core scanning, the MSQ1 coal rock shows high grayscale, which is indicative of high clay content.

The bottom boundary (SB2) of the MSQ2 is a transition surface where water depth changes from shallow to deep, while its top boundary (SB3) is a lithologic transition surface from mudstone (indicating relatively deep water) to coal seam (indicating relatively shallow water). Except for the low V/I ratios in the coal seam near the mudstone interlayer, the V/I ratios of the entire coal seam show a gradual increasing trend—this suggests that the MSQ2 formed in a sedimentary setting of slow sea-level rise. The grayscale model indicates calcite development in the MSQ2, and the grayscale value of the coal rock is lower than that of the MSQ1, signifying a decrease in clay content.

The top boundary (SB4) of the MSQ3 is a transition interface indicating deepening water, as indicated by the V/I ratio. The V/I ratios of the MSQ3 show a decreasing trend, which implies that the MSQ3 formed in a sedimentary setting of falling sea level (shallowing water).

The top boundary (SB5) of the MSQ4 is a transition interface indicating shallowing water, based on the V/I ratio. Although the V/I ratio exhibits local fluctuations, it shows an overall increasing trend. The grayscale model reveals the lowest grayscale value of coal rock in this interval, indicating extremely low clay content.

The top boundary (SB6) of the MSQ5 is a lithologic transition surface from coal rock to limestone. The V/I ratios in the coal seam gradually decrease, and the grayscale value of the coal rock is higher than that of the MSQ4.

Overall, the results of the intra-coal seam sequence division method proposed in this study show good consistency with the characteristics of the 3D grayscale model constructed from full-diameter core scanning—this fully confirms the high reliability of the intra-coal seam sequence division method developed in this research.

### Sequence stratigraphic distribution characteristics

Based on the aforementioned single-well fifth-order sequence division principles, this study conducted sequence stratigraphic division for the No. 8 coal seam across 47 wells within the study area. One northeast-trending profile and one southeast-trending profile in the study were selected for analysis to establish a fifth-order sequence stratigraphic framework for the No. 8 coal seam in the Daning-Jixian Block. This framework clarifies the spatial distribution characteristics of the fifth-order sequences in the No. 8 coal seam, laying a foundation for subsequent research on coal facies distribution patterns and coal accumulation patterns within the sequence stratigraphic framework.

#### Northeast-trending sequence stratigraphic framework and distribution characteristics

**Fig. 8:**
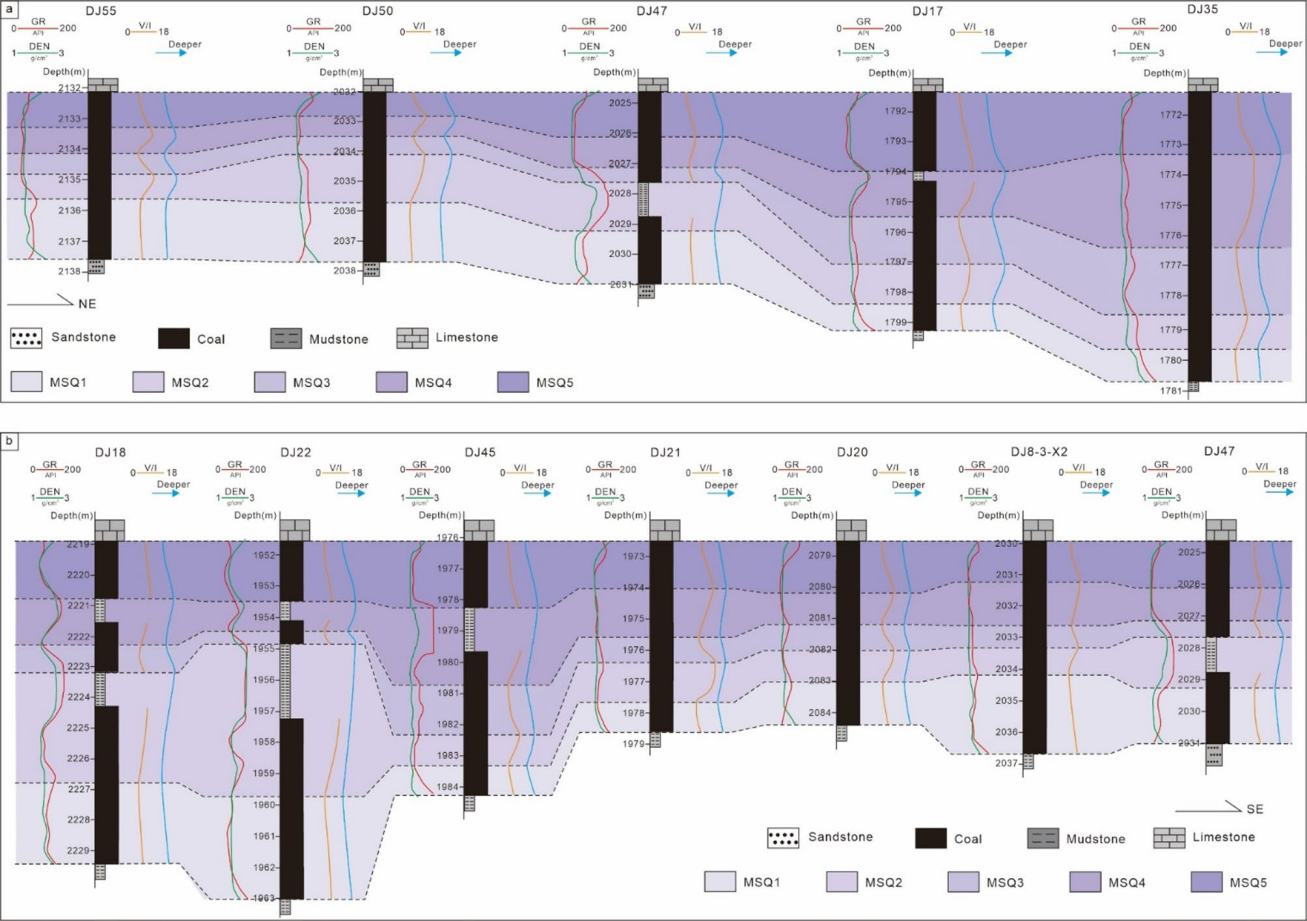
Microsequence (fifth-order sequence) correlation section of the No. 8 coal seam. (**a**) Northeast-trending well section (DJ55-DJ50-DJ47-DJ17-DJ35); section location shown in Fig. 1b (AA’). (**b**) Southeast-trending well section (DJ18-DJ22-DJ45-DJ21-DJ20-DJ8-3-X2-DJ47); section location shown in Fig. 1b (BB’).

The connecting-well section DJ55–DJ50–DJ47–DJ17–DJ35 extends from southwest to northeast in the study area (Figs. 1b and 8a). The No. 8 coal seam is stably developed, with a thickness ranging from 6 to 10 m across this section. The thickness of the MSQ1 shows a distribution characteristic of thinning toward the northeast. The MSQ2 is relatively thick in the Well DJ47 and DJ50 areas, while its thickness remains stable in other well area. The sedimentary center of the MSQ3 is located in the Well DJ17 and DJ35 areas, with a thickness of nearly 2 m. The MSQ4 is stably developed, with a relatively greater thickness in the Well DJ35 area. In other well areas, MSQ4 develops more uniformly without significant thickness variation. The sedimentary centers of the MSQ5 are located in the Well DJ17 and DJ50 areas, where its thickness exceeds 2.5 m. In other well areas, the thickness of the MSQ5 ranges from 1.5 to 2 m.

#### Southeast-trending sequence stratigraphic framework and distribution characteristics

The selected southeast-trending connecting-well section extends from northwest to southeast, passing through wells DJ18, DJ22, DJ45, DJ21, DJ20, DJ8-3-X2, and DJ47 (Figs. 1b and 8b). The thickness of the No. 8 coal seam, including mudstone interlayers, gradually decreases toward the southeast. The sedimentary centers of the MSQ1 and MSQ2 are located in the Well DJ18 and DJ22 areas in the northwest, where their thicknesses are relatively large. Moving southeastward, their thicknesses gradually decrease. The MSQ3 is stably developed across the entire southeastward section with a relatively small thickness. The sedimentary center of the MSQ4 shifts to the Well DJ45 area. Although mudstone interlayers also occur in the Well DJ18 and DJ21 areas, the thickness of the MSQ4 is relatively small. There is no significant change in the thickness of the MSQ4 in the well areas in the southeast. The thickness distribution of the MSQ5 shows no obvious spatial differentiation, with minimal thickness fluctuation, indicating a stable sedimentary environment during this period.

### Sequence-coal facies study

#### Coal facies distribution characteristics within the sequence stratigraphic framework

**Fig. 9:**
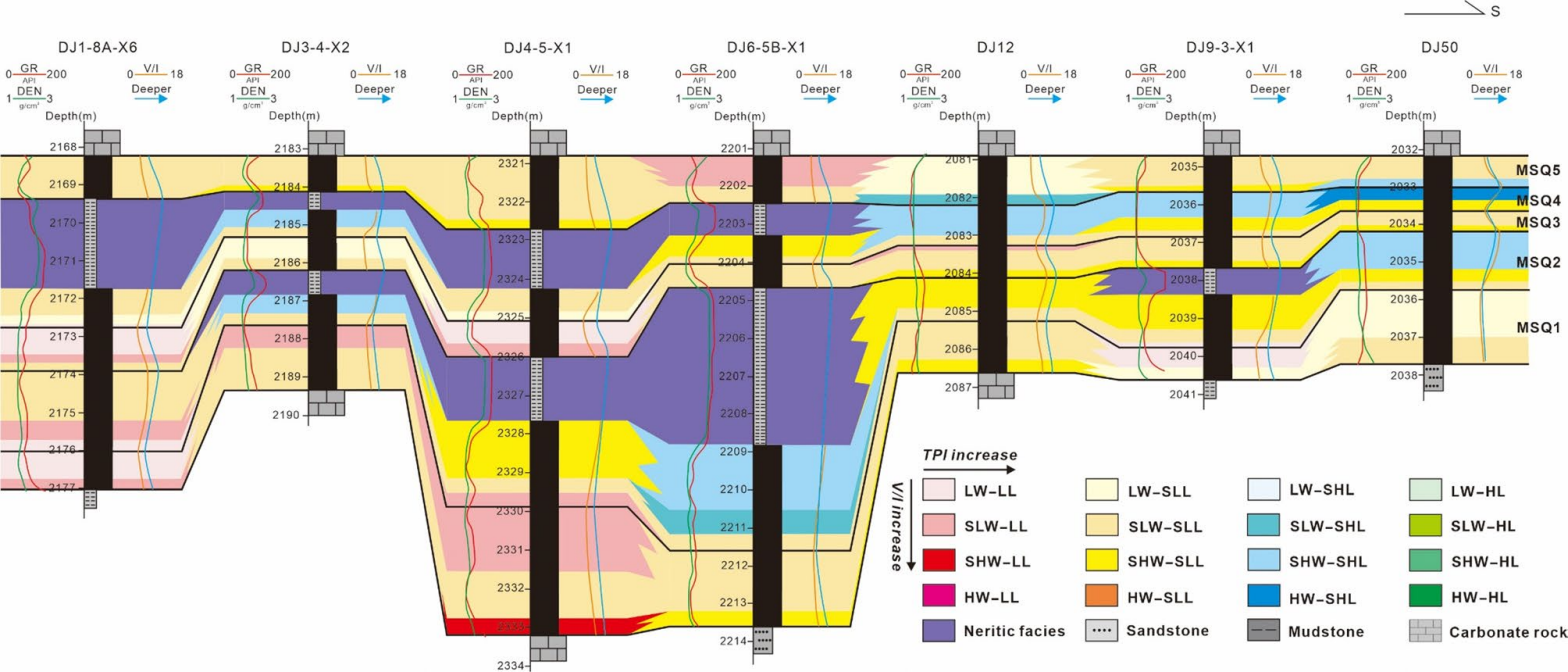
Correlation section of microsequence (fifth-order sequence) and coal facies within No. 8 coal seam through wells DJ1-8A-X6, DJ3-4-X2, DJ4-5-X1, DJ6-5B-X1, DJ12, DJ9-3-X1, and DJ50 (The section location is shown in Fig. 1(c) CC’).

Based on the calculated V/I ratios and TPI values of the No. 8 coal seam, and by comparing these metrics with the coal facies classification criteria of the Daning-Jixian Block defined in this study, the coal facies distribution characteristics within the sequence stratigraphic framework of the No. 8 coal seam were systematically analyzed. Taking a north-south trending connecting-well section as an example, this section extends from north to south, passing through Well DJ1-8 A-X6, DJ3-4-X2, DJ4-5-X1, DJ6-5B-X1, DJ12, DJ9-3-X1, and DJ50 (Figs. 1b and 9). The MSQ1 corresponds to the initial stage of peat swamp formation for the No. 8 coal seam, with a relatively thin thickness. From the perspective of sedimentary environment parameters, the water level of the coal-forming swamp is dominated by low water (LW) – slightly low water (SLW), and only local areas experience slightly high water (SHW) due to short-term water accumulation. This reflects weak hydrodynamic conditions and small spatial differences in the initial swamp environment. The density of ligneous plants is at the low (LL) – slightly low (SLL) level.

The sedimentary stage of the MSQ2 enters a progress of gradual water deepening with continuous expansion of regional accommodation space and significant changes in the sedimentary environment. Affected by water deepening, shallow marine mudstone interlayers appear in most well areas, which indirectly confirms the upward trend of the sedimentary base level during the period. Compared with the MSQ1, The water level of the coal-forming swamp is significantly higher, mainly characterized by slightly low water (SLW) – slightly high water (SHW), with both hydrodynamic conditions and reduction degree enhanced. With the optimization of the sedimentary environment, the growth conditions for ligneous plants are improved. The ligneous plant density is significantly higher than that in the MSQ1, and the input of plant residues increases. Additionally, the occurrence of argillaceous interlayers indicates that the coal facies are obviously affected by intermittent seawater.

During the deposition period of MSQ3, the regional water body is generally shallower, and the accommodation space is greatly reduced, resulting in limited sedimentation space for the coal seam. Thus, the thickness of the coal seam is significantly thinner than that of the MSQ2. Shallowing water is not conductive to the continuous growth and preservation of ligneous plants, so the density of ligneous plants is significantly lower than that in the MSQ2.

In the sedimentary stage of the MSQ4, the water body deepens again, and the regional sedimentary base level rises once more. A large section of muddy deposits appears in the northern regions of the study area, reflecting that the northern region is more significantly affected by the water body. In some well areas, such as Well DJ50, the swamp water level reaches high water (HW), and the reducing environment is further enhanced. Favorable hydrological and environmental conditions promote the growth of ligneous plants again, so the plant density increases once more compared with that in the MSQ3.

MSQ5 enters a regional water regression stage, and the sedimentary environment tends to be stable overall. The variation range of accommodation space decreases, and the sedimentary thickness of the coal seam in each well area is relatively consistent, with no obvious differences in thickness, which reflects that the sedimentary system has reached a state of dynamic equilibrium. During this period, the swamp water level is mainly slightly low water (SLW) – slightly high water (SHW), and the density of ligneous plants is mainly at the slightly low (SLL) level, with uniform spatial distribution and no significant regional differences.

#### Coal facies plane distribution characteristics

Based on lithological data reconstructed from logging curves and well log records, this study statistically analyzed the stratigraphic thicknesses and net coal thicknesses of each fifth-order sequence within the No. 8 coal seam across 47 wells in the Daning-Jixian Block. Additionally, the SSA-SVR model was used to calculate the average TPI values and V/I ratios for each fifth-order sequence, and contour maps of stratigraphic thickness, net coal thickness, TPI values, and V/I ratios were compiled for the No. 8 coal seam. With contour maps of stratigraphic thickness, net coal thickness, V/I ratio, and TPI values as boundary constraints, and integrating lithologic control factors while fully considering the rationality of coal facies development, coal facies plane maps for the MSQ1, MSQ2, MSQ3, MSQ4, and MSQ5 were compiled by analyzing the dominant facies within the fifth-order sequence framework. It is particularly noted that this study defined that if the ratio of the thickness of mudstone interlayers to the thickness of the sequence stratigraphy within a single microsequence is greater than 0.5, the microsequence is dominated by shallow marine deposits.

**Fig. 10:**
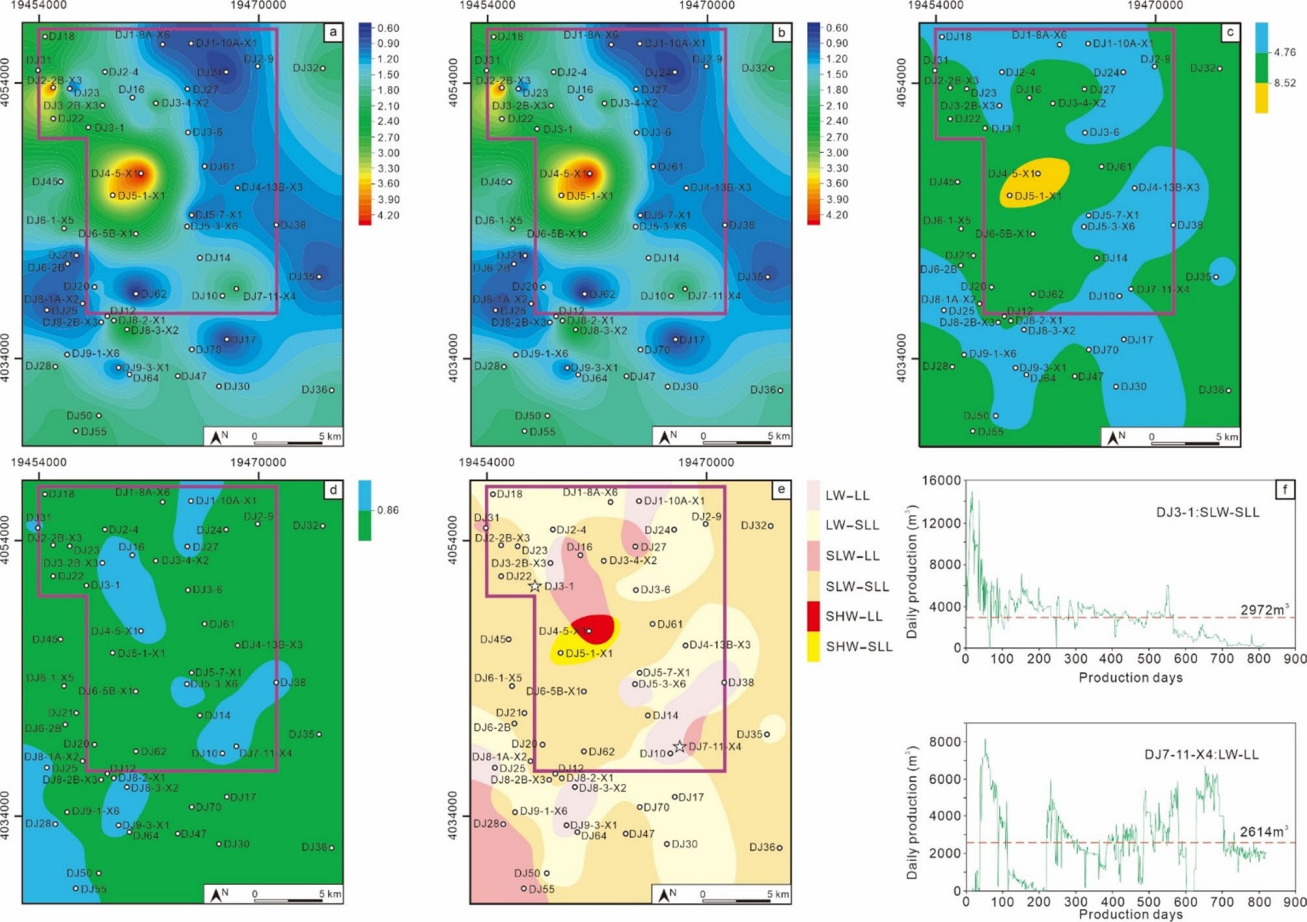
Planar distribution characteristics of the MSQ1. Contour map of sequence thickness (**a**), contour map of net coal thickness (**b**), contour map of V/I ratios (**c**), contour map of TPI values (**d**), coal facies distribution map (**e**), and production curves of typical production wells (**f**).

The MSQ1 corresponds to the initial swamping stage of the No. 8 coal seam. No muddy sediments are developed across the entire area, so the sequence thickness is consistent with the net coal thickness (Fig. 10a and b). During this period, the coal-forming sedimentary center was located in the northwest. The V/I ratio contour map also indicates that the water depth was relatively deep in the central and northwestern parts of the block (Fig. 10c). The TPI contour map shows that the density of ligneous plants was relatively low, dominated by low density (LL)–slightly low density (SLL) (Fig. 10d). The coal facies plane map shows that during the MSQ1 stage, the study area was mainly dominated by two coal facies types: slightly low water–slightly low density ligneous plants (SLW-SLL) and low water–slightly low density ligneous plants (LW-SLL) (Fig. 10e). Two typical wells in the study area with MSQ1 as their production interval, Well DJ3-1 and Well DJ7-11-X4, were selected for productivity analysis (Fig. 10f). Well DJ3-1 is developed with the SLW-SLL facies, with a cumulative production period of 813 days and an average daily gas production of 2972 m³. Its production characteristics show high yield in the early stage and low yield in the later stage. Well DJ7-11-X4 is developed with the low water – low density ligneous plants (LW-LL) facies, with a cumulative production period of 818 days and an average daily gas production of 2614 m^3^. It initially had no gas production; after a period of production, the gas production could reach over 6000 m^3^, but its stable production capacity is poor.

**Fig. 11:**
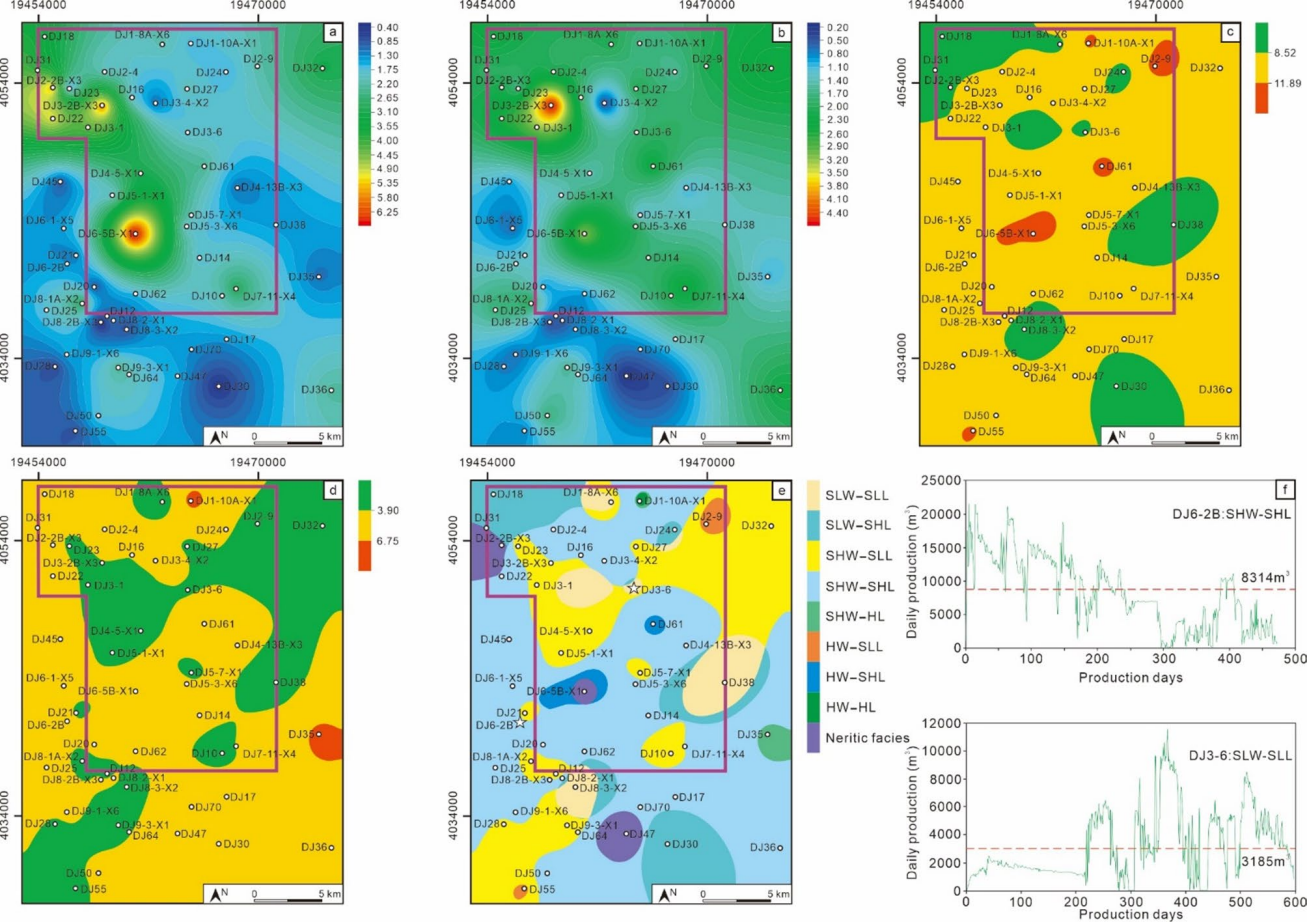
Planar distribution characteristics of the MSQ2. Contour map of sequence thickness (**a**), contour map of net coal thickness (**b**), contour map of V/I ratios (**c**), contour map of TPI values (**d**), coal facies distribution map (**e**), and production curves of typical production wells (**f**).

The MSQ2 entered the first transgression stage. Affected by seawater intrusion, shallow marine argillaceous deposits developed in certain areas, leading to a significant difference between stratigraphic thickness and net coal thickness (Fig. 11a and b). The V/I ratio contour map shows that most areas of the block were in a slightly high water (SHW) environment at this time, with some local areas even reaching high water (HW) (Fig. 11c). The density of ligneous plants was noticeably higher than that in MSQ1, dominated by slightly low density (SLL) – slightly high density (SHL), indicating a marked improvement in vegetation growth conditions. The coal facies plane map indicates that shallow marine deposits existed in some areas during the MSQ2 stage, which reflects that the rate of water intrusion was relatively fast, disrupting the continuous sedimentation of swamp peat. Overall, the coal facies were mainly slightly high water – slightly high density ligneous plants (SHW-SHL) and slightly high water – slightly low density ligneous plants (SHW-SLL). This suggests that the water depth was relatively deep during this period, which not only provided sufficient moisture for plant growth but also created a favorable reducing environment for preservation—both the input and preservation conditions of plant residues were relatively optimal. The main production intervals of Well DJ6-2B and Well DJ3-6 are MSQ2. Well DJ6-2B belongs to the SHW-SHL facies, while Well DJ3-6 belongs to the SLW-SLL facies. Well DJ6-2B has a cumulative production period of 471 days, with an average daily gas production of 8314 m^3^, and it exhibited high initial gas production capacity, but after 250 days of production, the daily gas production began to fall below the average. Well DJ3-6 currently has a cumulative production period of 599 days, with an average daily gas production of 3185 m^3^. The significant difference in productivity between the two wells directly confirms the key controlling effect of the coal-forming environment on the productivity of coalbed methane wells.

**Fig. 12:**
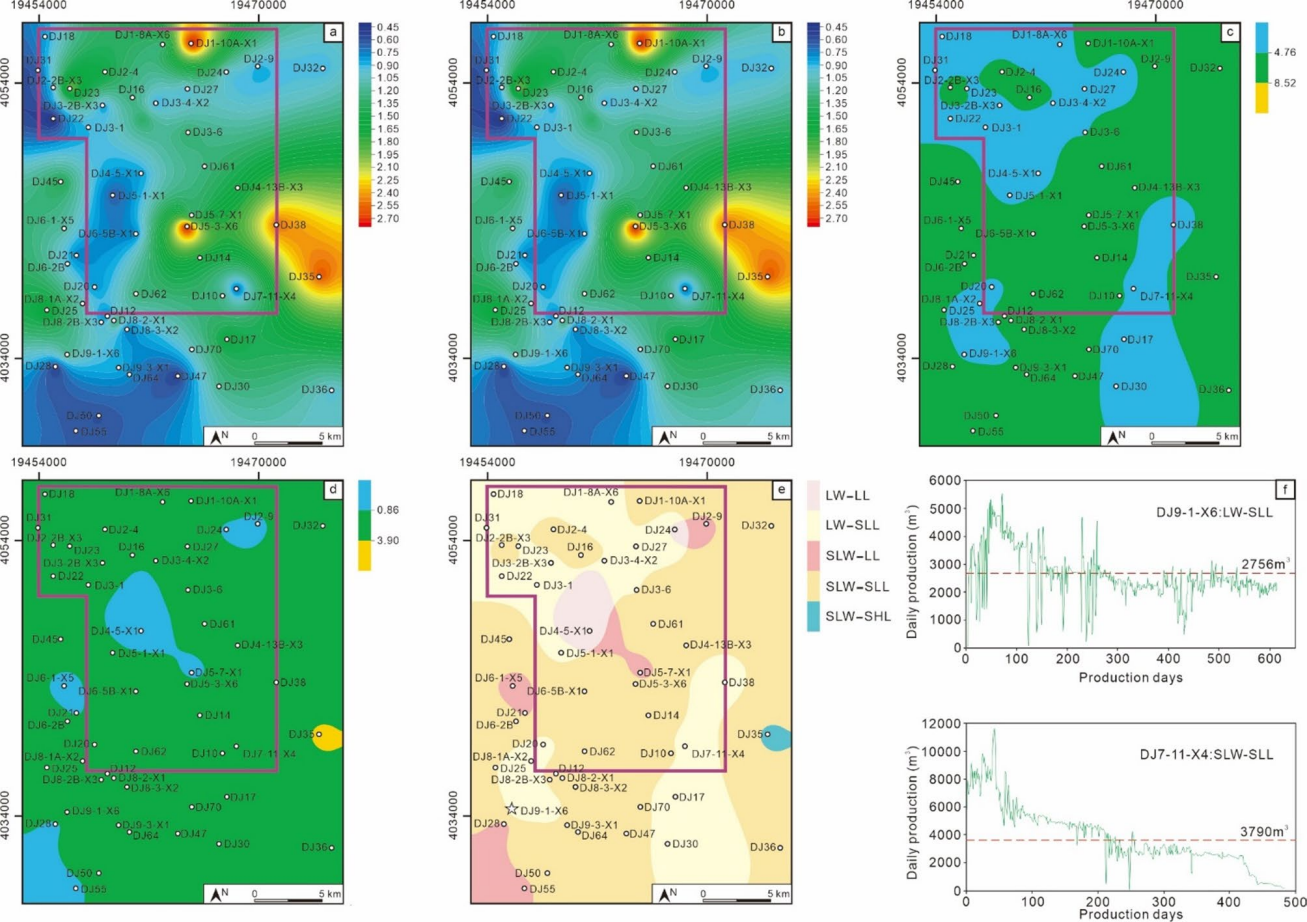
Planar distribution characteristics of the MSQ3. Contour map of sequence thickness (**a**), contour map of net coal thickness (**b**), contour map of V/I ratios (**c**), contour map of TPI values (**d**), coal facies distribution map (**e**), and production curves of typical production wells (**f**).

The MSQ3 was deposited during a water regression stage. The entire area was a swamp environment, with no argillaceous deposits developed. Thus, the sequence thickness was completely consistent with the net coal thickness being completely consistent (Fig. 12a and b). During this period, the water environment across the block was dominated by low water (LW) – slightly low water (SLW) (Fig. 12c), and the density of ligneous plants was also relatively low, primarily falling into the low density (LL) – slightly low density (SLL) range (Fig. 12d). The coal-forming environments were mainly slightly low water – slightly low density ligneous plants (SLW-SLL) and low Water – slightly low density ligneous plants (LW-SLL) (Fig. 12e). Two wells with MSQ3 as their main production interval, Well DJ9-1-X6 and Well DJ7-11-X4, were selected for productivity comparison. Well DJ9-1-X6 is characterized by the LW-SLL facies. It has a cumulative production period of 614 days, with an average daily gas production of 2756 m^3^. While it exhibits relatively good gas production stability, its overall production is relatively low. Well DJ7-11-X4 is characterized by the SLW-SLL facies. It has a cumulative production period of 482 days, with an average daily gas production of 3780 m^3^. Since the perforation interval of this well partially covers MSQ3 and is influenced by the superposition of high-quality coal facies, its gas production performance is higher than that of Well DJ3-1 (which takes MSQ1 as its production interval and also belongs to the SLW-SLL environment).

**Fig. 13:**
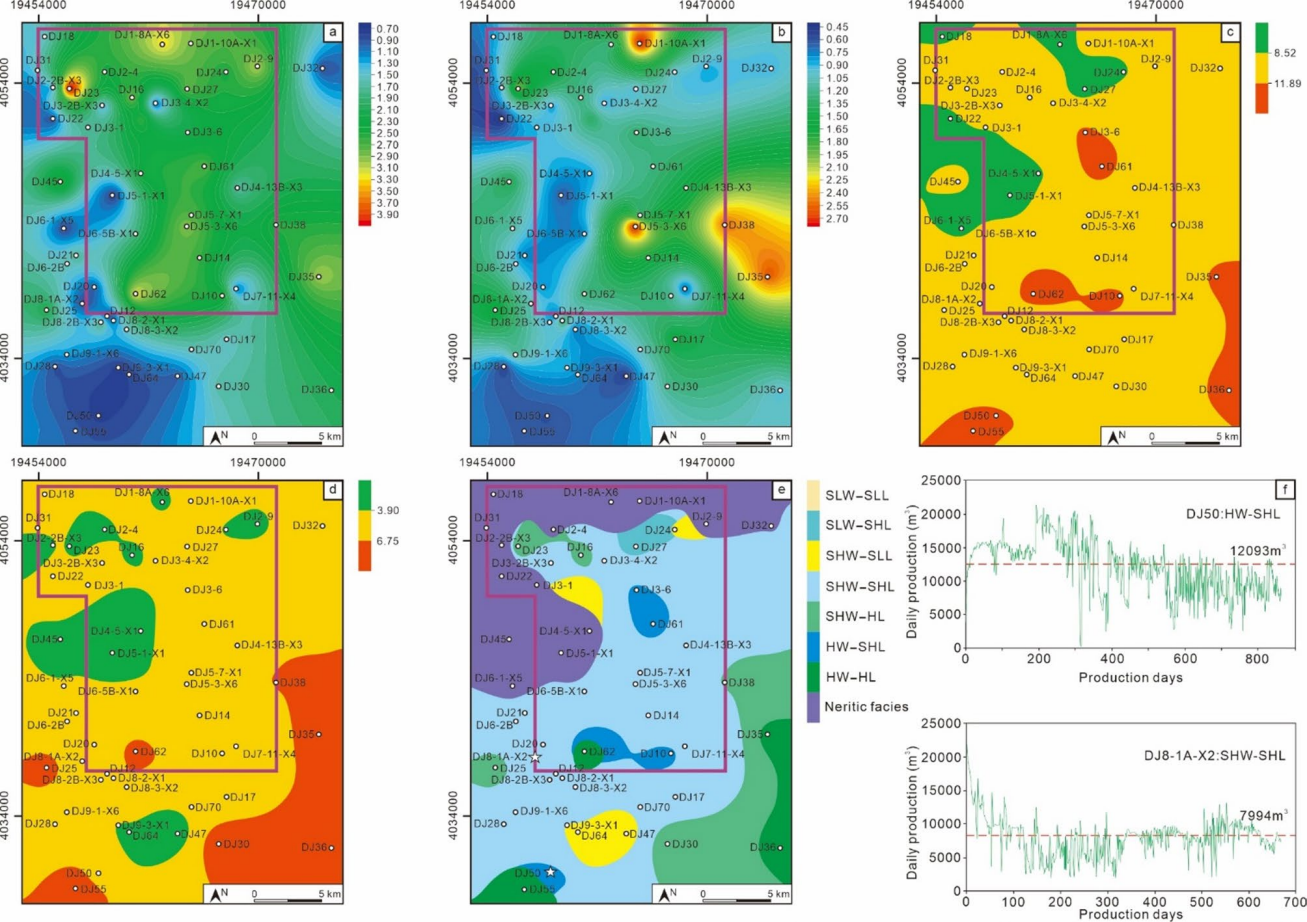
Planar distribution characteristics of the MSQ4. Contour map of sequence thickness (**a**), contour map of net coal thickness (**b**), contour map of V/I ratios (**c**), contour map of TPI values (**d**), coal facies distribution map (**e**), and production curves of typical production wells (**f**).

The MSQ4 corresponds to the second transgression stage, with a more significant transgression intensity than that of MSQ2. The northern part of the study area shows a notable difference between sequence thickness and net coal thickness (Fig. 13a and b), indicating that shallow marine argillaceous deposits are widely developed in the north. The dominant water environment was slightly high water (SHW) – high water (HW), and the density of ligneous plants was mainly slightly high density (SHL) – high density (HL). However, the water environment in the northern and northwestern regions was slightly low water (SLW), with ligneous plant density at slightly low density (SLL). This may be due to the development of argillaceous deposits in these areas; concurrently, the thin net coal thickness resulted in relatively lower calculated coal facies parameters in the coal seam compared to other locations. The coal facies plane map indicates that large-scale shallow marine deposits developed in the northern part of the study area, reflecting that the transgression during this period was characterized by high speed and a wide range, leading to a higher rate of accommodation space increase than peat accumulation rate in the peat swamp, which inhibited the continuous sedimentation of peat. In the remaining areas, the main coal facies are slightly high water – slightly high density ligneous plants (SHW-SHL) and slightly high water – high density ligneous plants (SHW-HL), making this interval the main development zone for high-quality coal facies in the No. 8 coal seam.

Both Well DJ50 and Well DJ8-1 A-X2 have MSQ4 as their main production interval. Well DJ50 is characterized by the high water – slightly high density ligneous plants facies (HW-SHL). It has a cumulative production period of 863 days, with an average daily gas production of 12,093 m^3^, and maintained daily production above 10,000 m^3^ for 602 days, demonstrating strong high-yield and stable production capabilities. Well DJ8-1 A-X2 belongs to the slightly high water – slightly high density ligneous plants facies (SHW-SHL). It has a cumulative production period of 668 days, with an average daily gas production of 7,994 m^3^, and exhibits relatively good stable production performance.

**Fig. 14:**
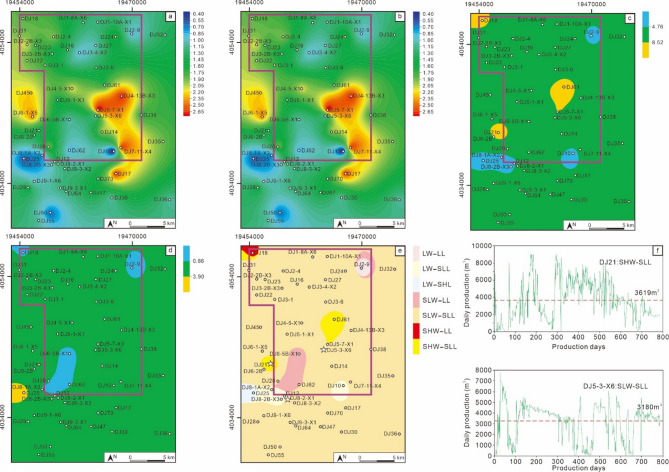
Planar distribution characteristics of the MSQ5. Contour map of sequence thickness (**a**), contour map of net coal thickness (**b**), contour map of V/I ratios (**c**), contour map of TPI values (**d**), coal facies distribution map (**e**), and production curves of typical production wells (**f**).

During the sedimentary period of MSQ5, the study area entered a regional water regression stage. Compared with the MSQ3, the water retreat was slower and the sedimentary environment was more stable, characterized by larger sequence thickness and net coal thickness than those in the MSQ3 (Fig. 14a and b). A contradiction was identified when comparing the net coal thickness contour map and the V/I ratio contour map (Fig. 14b and c). Traditionally, it is believed that a larger net coal thickness indicates a deeper swamp water during the coal-forming period. The key factor leading to a thicker coal seam is the consistency between the rate of accommodation space increase and the peat accumulation rate (Shao et al., 2009; Wang et al., 2018). However, during the sedimentary period of the MSQ5, the V/I ratio did not increase correspondingly in areas with larger net coal thickness. This study proposes that during the MSQ5 deposition period, the peat swamp mainly inherited the high water level from the MSQ4 deposition period. With the gradual retreat of swamp water, the water environment entered a semi-oxidizing state, which is unfavorable for the preservation of vitrinite, thereby resulting in a decrease in the V/I ratio (Fig. 14c). TPI values indicate that the density of ligneous plants was dominated by slightly low density (SLL), with local low density (LL) (Fig. 14d). The coal facies plane map shows that the study area was mainly dominated by the slightly low water – slightly low density ligneous plants coal facies (SLW-SLL) (Fig. 14e). Typical wells with MSQ5 as their production interval were selected for analysis. Well DJ21 is in the slightly high water – slightly low density ligneous plants environment (SHW-SLL), with a cumulative production period of 784 days and an average daily gas production of 3619 m^3^. Well DJ5-3-X6 is in the slightly low water – slightly low density ligneous plants environment (SLW-SLL), with a cumulative production period of 787 days and an average daily gas production of 3180 m^3^. When the density of ligneous plants is similar, subtle changes in water level can also affect the productivity of coalbed methane wells.

**Fig. 15:**
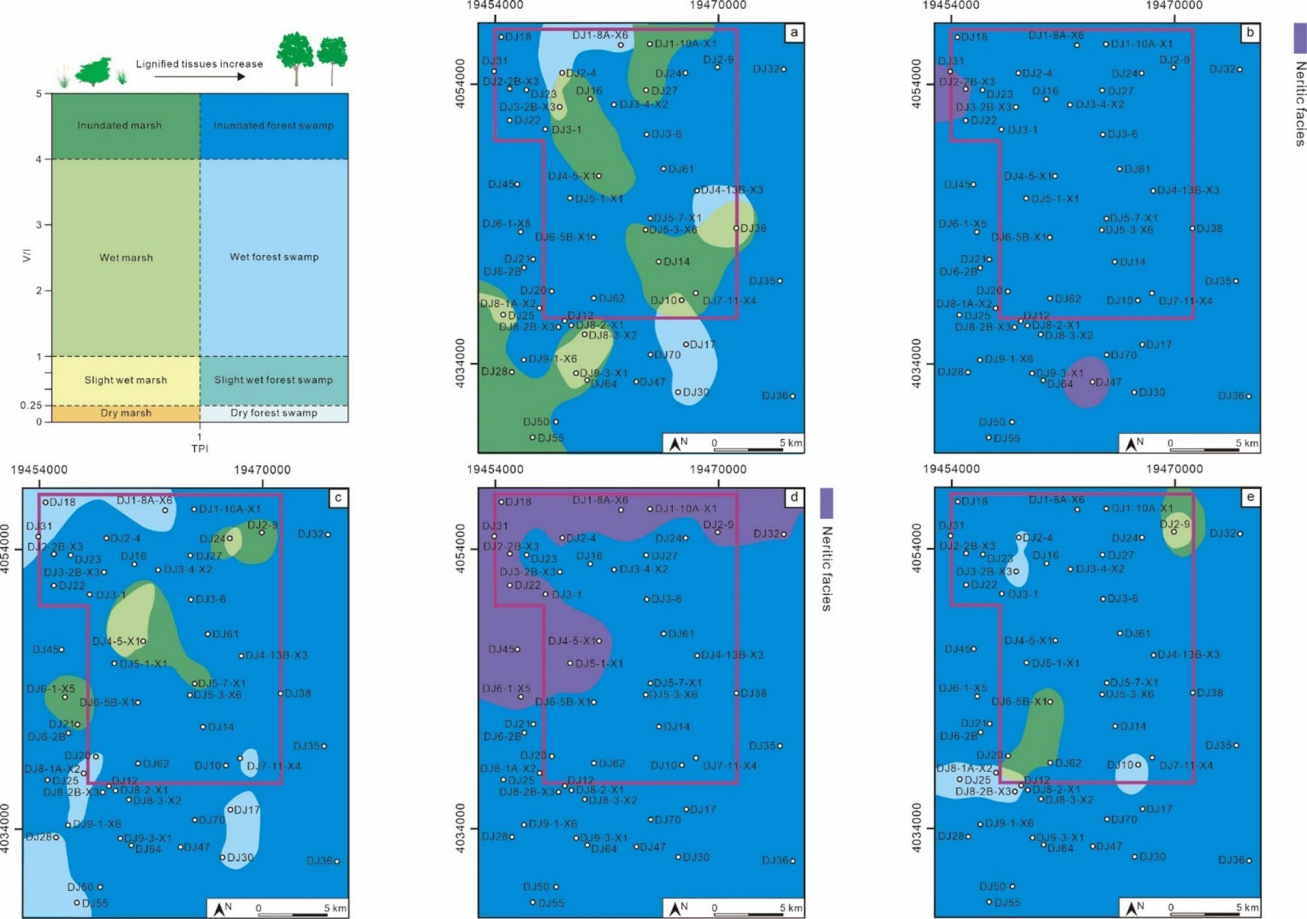
Coal facies plane maps based on the traditional scheme. The traditional classification scheme (Yuan and Li, 2015; Lamberson et al., 1991) (a); coal facies map of MSQ1 (**b**); coal facies map of MSQ2 (**c**); coal facies map of MSQ3 (**d**); coal facies map of MSQ4 (**e**); coal facies map of MSQ5 (**f**).

Based on the existing traditional schemes for V/I and TPI (Fang et al., 2003; Yuan & Li, 2015; Lamberson et al., 1991), a total of 8 coal facies types were combined and derived (Fig. 15a). According to this scheme, the MSQ1-MSQ5 were all dominated by inundated forest swamp (Fig. 15b and e); especially during the MSQ2 and MSQ4 stages, only inundated forest swamp developed across the entire block (Fig. 15c and e).

Compared with the coal facies classification scheme proposed in this study, the traditional scheme fails to accurately distinguish the coal facies differences among different microsequences, and it is even more difficult to establish a direct correlation with coalbed methane well productivity. Thus, it cannot provide effective guidance for the optimal selection of favorable areas, which highlights the practical value of the coal facies classification scheme proposed in this study.

## Conclusion

This study focuses on the No. 8 coal seam of the Taiyuan Formation in the Daning-Jixian Block on the eastern margin of the Ordos Basin, developed a coal facies parameter classification scheme applicable to the Daning-Jixian Block, and reconstructed the peat swamp environment during the deposition of the No. 8 coal seam. The main conclusions are as follows:

1. This study established logging response-based SSA-SVR models for calculating V/I ratios and TPI values. For the V/I-SSA-SVR model, the final optimized model achieved an unbiased MAPE of 18.73%, an unbiased RMSE of 1.36, and an unbiased R^2^ of 88.69%. For the TPI-SSA-SVR model, the optimal model achieved an unbiased MAPE of 17.90%, an unbiased RMSE of 1.12, and an unbiased R^2^ of 86.39%.
2. Cluster analysis was used to divide TPI values (which reflect the vegetation type of coal-forming swamps) into four categories: <0.86 represents low-density ligneous plants, 0.86–3.90 represents slightly low-density ligneous plants, 3.90–6.75 represents slightly high-density ligneous plants, and > 6.75 represents high-density ligneous plants. V/I ratios (which reflect the water depth of coal-forming swamps) were also divided into four categories: <4.76 represents low water, 4.76–8.52 represents slightly low water, 8.52–11.899 represents slightly high water, and > 11.89 represents high water. By combining these two parameters, a 16-category coal facies classification scheme suitable for the Daning-Jixian Block was ultimately developed.
3. This study used water depth transition interfaces and lithological transition interfaces as sequence boundaries to divide microsequences (fifth-order sequences) within coal seams, and established an isochronous stratigraphic framework for the No. 8 coal seam in the Daning-Jixian Block. Based on the established coal facies classification scheme, we reconstructed the peat swamp environment during the deposition of each fifth-order sequence, providing a reference for subsequent deep coalbed methane exploration and development.

## Supplementary Information

Below is the link to the electronic supplementary material.

Supplementary Material 1

## Data Availability

Data is available within supplementary information files.

## Abbreviations

AC: Acoustic log (µs/m)
CN: Neutron porosity log (dec)
DEN: Density log (g/cm^3^)
GR: Gamma ray log (API)
HL: High density ligneous plants
HW: High water
LL: Low density ligneous plants
LW: Low water
RT: Resistivity log (Ω)
SHL: Slightly high density ligneous plants
SHW: Slightly high water
SLL: Slightly low density ligneous plants
SLW: Slightly low water
SP: Spontaneous potential log (mV)
SSA: Sparrow search algorithm
SVR: Support vector regression
SSA-SVR: Support vector regression model optimized sparrow search algorithm
TPI: Tissue preservation index
TPI-SSA-SVR: Support vector regression model optimized sparrow search algorithm for calculating TPI
V/I: Vitrinite-inertinite ratio
V/I-SSA-SVR: Support vector regression model optimized sparrow search algorithm for calculating V/I
